# Deletion of miR‐122‐5p Exacerbated Hyperthyroidism‐Induced Liver Injury by Regulating Ferroptosis

**DOI:** 10.1155/ije/7047803

**Published:** 2026-06-23

**Authors:** Guoxian Zhu, Lidan Zhang, Wenbao Huang, Jiexia Ding, Lifei Yu, Xiangfei Xu

**Affiliations:** ^1^ Department of Infectious Diseases, School of Medicine, Affiliated Hangzhou First People’s Hospital, Westlake University, Hangzhou, Zhejiang, China, westlake.edu.cn

**Keywords:** CCDC6, ferroptosis, hyperthyroidism, liver damage, miR-122-5p

## Abstract

The aim of this study was to evaluate the effect and mechanism of miR‐122‐5p on hepatic injury induced by hyperthyroidism. Male Sprague Dawley rats were divided into three groups: a normal thyroid function group (sham), a levothyroxine (LT4)–induced hyperthyroidism group (T4 sodium), and a hyperthyroidism group treated with a miR‐122‐5p inhibitor (T4 sodium + antagomir‐122‐5p). Liver tissue pathology, ferroptosis and inflammatory markers, and miR‐122‐5p expression were assessed. Finally, rat hepatocytes (BRL‐3A) were treated with carbon tetrachloride (CCL4) to explore the role of the miR‐122‐5p/CCDC6 axis in hepatocyte injury. We found that miR‐122‐5p was expressed at low levels in liver tissues from hyperthyroid rats. Inhibition of miR‐122‐5p aggravated liver tissue injury. Additionally, in hyperthyroid rats, inflammatory factors, iron content, and markers of ferroptosis were upregulated by miR‐122‐5p inhibitors. Mechanistically, miR‐122‐5p was found to bind to CCDC6 and negatively regulate CCDC6 expression. Furthermore, miR‐122‐5p deletion induced hepatocyte injury by exacerbating the ferroptosis process and mitochondrial damage in CCL4‐treated hepatocytes, a process dependent on CCDC6 expression. The miR‐122‐5p/CCDC6 axis plays a protective role in hyperthyroidism‐induced liver injury and serves as a potential target for the clinical treatment of hyperthyroidism‐associated liver disease.

## 1. Introduction

Thyroid hormones play crucial roles in development, metabolism, thermoregulation, and growth [1, 2]. However, in pathological conditions, hyperthyroidism results from an overactive thyroid gland, leading to excessive production and secretion of thyroid hormones [3, 4]. Hyperthyroidism affects multiple body systems, including the nervous, cardiovascular, and gastrointestinal systems [5, 6]. The liver is a key organ influenced by thyroid hormones, and their overproduction can cause oxidative damage to the liver [7, 8]. Consequently, patients with hyperthyroidism often experience liver dysfunction. Nevertheless, the mechanisms underlying liver injury caused by hyperthyroidism require further investigation.

Ferroptosis is an iron‐dependent form of cell death that is distinct from other types of cell death, such as apoptosis, autophagy, and cellular pyroptosis. The primary mechanism of ferroptosis involves the catalysis of lipid peroxidation of unsaturated fatty acids, which reduces glutathione peroxidase 4 (GPX4) levels, thereby inducing cell death [9–11]. Consequently, the occurrence of ferroptosis is accompanied by disturbances in intracellular iron content and a significant increase in reactive oxygen species (ROS) [12, 13]. Ferroptosis plays a role in the pathogenesis of various liver diseases, nonalcoholic steatohepatitis, liver fibrosis, cirrhosis, and hepatocellular carcinoma [14, 15]. Moreover, oxidative stress is elevated in hepatic tissues under hyperthyroid conditions, with the expression of antioxidant enzymes, such as superoxide dismutase (SOD), catalase (CAT), and GPX, downregulated in mouse models of hyperthyroidism [16]. This suggests that large amounts of ROS may accumulate in liver tissue affected by hyperthyroidism. Additionally, the liver is the primary site for iron storage in the body, and liver injury is closely associated with iron overload [17]. Taken together, these findings suggest that hyperthyroidism may promote ferroptosis in liver tissue.

Notably, microRNAs (miRNA/miR) play a key role in regulating gene expression. miRNAs are small endogenous RNAs that regulate many physiological and pathological processes by controlling gene expression at the post‐transcriptional level [18]. In hyperthyroidism, miRNAs also play an important role. For example, miR‐29a‐3p negatively regulated circulating Tfh memory cells in Graves′ disease (GD) patients by targeting ICOS [19]. MiR‐221 is a member of the miRNA family, primarily classified into the miR‐221‐3p and miR‐221‐5p subtypes. Studies have shown that miR‐221 expression is often dysregulated in thyroid diseases. For instance, miR‐221‐3p expression tends to be downregulated in hypothyroidism [20]. Similarly, miR‐221‐5p is abnormally expressed in the serum of patients with thyroid dysfunction, such as GD [21]. Additionally, miR‐221 is closely associated with liver injury processes. In hepatitis B virus (HBV)–infected liver tissues, miR‐221‐3p levels are elevated and may target genes involved in innate immune responses and viral replication [22]. Meanwhile, miR‐221‐5p is linked to various liver abnormalities, including drug‐induced liver dysfunction [23, 24]. Furthermore, upregulation of miR‐122‐5p has been observed in patients with hepatitis [25]. However, whether miR‐221 influences hyperthyroidism‐induced liver injury remains unclear and warrants further in‐depth investigation.

This study centered on the role and mechanism of miRNA‐122‐5p in hyperthyroidism‐induced liver injury. It was found that inhibition of miRNA‐122‐5p aggravated hyperthyroidism‐induced ferroptosis and inflammation to induce liver injury by regulating coiled‐coil domain‐containing 6 (CCDC6) expression.

## 2. Materials and Methods

### 2.1. Animals

Seven‐week‐old male Sprague Dawley rats were obtained from the Shanghai Laboratory Animal Center (China). They were in a moisture‐controlled room (50%–70%) with a 12‐h light–dark cycle and free access to food. All animal procedures were approved by the Affiliated Hangzhou First People’s Hospital Ethics Committee (HZSY2024033‐1) and conformed to the standards outlined in the Guide for the Ethical Care and Use of Laboratory Animals.

### 2.2. Animal Model and Treatment

After one week of acclimatization, the rats were randomly divided into six groups: sham‐operated group (Sham, *n* = 6), hyperthyroidism group (T4 sodium, *n* = 6), and the group of hyperthyroidism rats treated with miR‐122‐5p inhibitor (T4 sodium + antagomir‐122‐5P, *n* = 6). To induce hyperthyroidism, rats were administered L‐thyroxin (LT4, #HY‐18341B, MedChemExpress, USA) at 1 mg/kg/d by gavage for 10 days, and the sham group was treated with an equal volume of saline by gavage for 10 days. 5 nmol/day miR‐122‐5p inhibitor (antagomir‐122‐5P, #miR40000827‐4‐5, RiboBio, China) was injected through the tail vein for 3 days after the start of modeling. After 10 days, mice were decapitated and euthanized, and livers were obtained for analysis.

### 2.3. Histological Analysis

Livers were fixed in 10% neutral formalin, and paraffin‐embedded specimens were serially sectioned (5 μm) and stained with hematoxylin and eosin (H&E) (Beyotime, China). Each section was then examined for pathologic changes using a light microscope (Olympus BX‐50, Olympus Optical, Japan).

### 2.4. Immunohistochemistry (IHC)

Liver tissues were fixed with 4% paraformaldehyde at 4°C overnight and paraffin‐embedded. Sections were then deparaffinized, rehydrated, antigenically repaired, treated with 3% hydrogen peroxide, permeabilized with 0.2% Triton X‐100/PBS, and blocked with 1% BSA. Sections were then incubated with CCDC6 antibody (#PA5‐53905, 1:200 dilution, Thermo, USA) at 4°C overnight. After washing, the sections were incubated with Goat Anti‐Rabbit IgG (H + L) HRP (#S0001, 1:200 dilution, Affinity, USA) for 1 h at room temperature. Finally, the sections were photographed with an IX73 inverted microscope (Olympus BX‐50, Olympus Optical, Japan).

### 2.5. Quantitative Reverse Transcription PCR (RT‐qPCR)

Rat liver tissues were subjected to RNA extraction using Mini RNA Isolation IITM (Zymo Research Corp, USA). Trizol reagent (TaKaRa Biotech, Japan) was used in the cells to extract RNA. First‐strand cDNA was synthesized from total RNA by RT using QuantiTect Reverse Transcription Kit (Qiagen, Germany) or from small RNA using miScript II RT Kit (Qiagen, Germany). SYBR Green Master Mix (Vazyme, China) was used for the qPCR. Quantification of gene expression was carried out using the 2^−ΔΔCt^ technique. MiR‐6837‐3p was normalized to U6. Primer sequences were as follows: miR‐122‐5p (Forward: 5′‐CGATACAGAGAATTAGCATGGC‐3′, Reverse: 5′‐CCTGGAGTGTGACAATGGTGTTTG‐3′); U6 (Forward: 5′‐CGATACAGAGAAGATTAGCATGGC‐3′, Reverse: 5′‐AACGCTT CACGAATTTGCGT‐3′); CCDC6 (Forward: 5′‐GGTAAATCGGCTGTGGAAG‐3′, Reverse: 5′‐TATGCCTCATCATGTTCTCG‐3′); GAPDH (Forward: 5′‐TGGAGTCTACTGGCGTCTT‐3′, Reverse: 5′‐GCTGACAATCTTGAGGGAG‐3′).

### 2.6. Enzyme‐Linked Immunosorbent Assay (ELISA)

Cells or sera from different groups were collected. Iron content was measured using an Iron Assay Kit (Colorimetric) (#ab83366, Abcam, UK). Inflammation factor TNF‐α and IL‐10 levels were detected using Rat TNF‐α ELISA Kit (#EK382/3–48, Mlbio, Shanghai, China) and Rat IL‐10 ELISA Kit (#EK310/2‐48, Mlbio, Shanghai, China) according to the manufacturer’s instructions. The optical density (OD) at 450 nm was assessed using a microplate reader (Thermo Fisher, Waltham, MA, USA).

### 2.7. Western Blot (WB) Analysis

Cells or tissues were lysed in RIPA buffer (50 mM Tris‐HCl, 150 mM NaCl, 1% NP‐40, 0.5% sodium deoxycholate, and 0.1% SDS, pH 7.4) supplemented with 1 × protease inhibitor mixture, and then, the protein content of the lysates was quantified by BCA kit (Beyotime, China). Proteins were electrophoresed in 10% sodium dodecyl sulfate‐polyacrylamide gels (SDS‐PAGE) and then transferred onto polyvinylidene difluoride (PVDF, Millipore, MA, USA) membranes. The membranes were then closed with 5% skimmed milk for 1 h at room temperature and then incubated with primary antibodies at 4°C overnight. Each membrane was then tested with multiple primary antibodies and stored at 4°C overnight. Information on the primary antibodies used in the study is as follows: anti‐CCDC6 (1:2000, #PA5‐53905, Thermo, USA), anti‐ferritin (1:2000, #ab75973, Abcam, UK), anti‐GPX4 (1:2000, #ab240277, Abcam, UK), anti‐ACSL4 (1:10,000, #ab155282, Abcam, UK), and anti‐GAPDH (1:2000, #AF7021, Affinity, Changzhou, China). After three TBST washes, the membranes were incubated with HRP‐labeled secondary antibody (1:3000) for 1 h at room temperature.

The images were then obtained by scanning the blots. With the aid of a very sensitive ECL chemiluminescence detection kit, the tagged protein bands were examined.

### 2.8. Cell Culture and Treatment

The BRL‐3A rat hepatocytes were obtained from the CellBank of Chinese Academy of Sciences (Shanghai, China) and cultured in H‐DMEM (Gibco, USA) + 10% FBS+ 1% penicillin–streptomycin medium at 37°C, 5% CO2 conditions for culture. BRL‐3A cells were treated with 50 μM carbon tetrachloride (CCL4, Aladdin, China) for 24 h to induce hepatocyte injury.

MiR‐122‐5p mimics or inhibitor and NC‐mimics or NC inhibitor were constructed by GeneChem (Shanghai, China). In addition, the shRNA targeting CCDC6 and the negative control shCtrl were also synthesized by GeneChem.293t or BRL‐3A cells were inoculated at 1.5 × 105 cells/well in 12‐well plates and then transfected with disordered miRNA (NC‐mimics/inhibitor, 20 nM), miR‐122‐5p mimics/inhibitor (20 nM), and shCCDC6 using Lipofectamine 2000 reagent (Invitrogen, USA) according to the manufacturer’s protocols.

### 2.9. Luciferase Reporter Assays

Wild‐type CCDC6 (CCDC6 3′UTR‐WT) and 3′‐UTR mutant plasmid of CCDC6 (CCDC6 3′UTR‐MUT) were synthesized by GeneChem (Shanghai, China), and then, these vectors were cotransfected with NC‐mimics or miR‐122‐5p mimics into BRL‐3A cells using Lipofectamine 2000 reagent (Invitrogen, USA). 48 h after transfection, luciferase activity was monitored using a dual‐luciferase reporter system (Promega, USA).

### 2.10. Cell Counting Kit 8 (CCK8)

Cells from the CCL4‐treated BRL‐3A cells (2000 cells/well) were placed in a 96‐well plate. Then, a solution of CCK8 (Beyotime Institute of Biotechnology, China) was added and left for 1 to 5 days. At various time points, the OD values were measured at 570 nm using a microplate reader (M2009PR, Tecan Infinite, Australia).

### 2.11. Flow Cytometry

BRL‐3A cells (2 × 105 cells/well) were inoculated in 6‐well plates and cultured for 24 h. Cells were then stained with Annexin‐V/PE Apoptosis Assay Kit (Thermo Fisher Scientific, USA) for 25 min at room temperature. Finally, stained cells were analyzed for apoptosis using a FACSCanto II flow cytometer (BD Biosciences, USA).

### 2.12. JC‐1 Assay

The JC‐1 test was used to identify the mitochondrial membrane potential. JC‐10 kits (Solarbio Science, China) were added to the cells and incubated for 20 min at 37°C, after which the cells were washed with 1 mL of PBS. After that, the cells were examined using a Zeiss Fluorescence Microscope (Germany) to examine the pictures of the intensity of the red and green fluorescence in each unit.

### 2.13. Statistical Analysis

All data were analyzed using GraphPad Prism software 8.0 (San Diego, USA) and presented as the mean ± standard deviation (SD). A one‐way ANOVA with post hoc Bonferroni corrections for multiple comparisons was used to identify statistically significant differences. The two groups were compared using the unpaired Student’s *t*‐test. Then, *p* < 0.05 was considered significant.

## 3. Results

### 3.1. Inhibition of miR‐122‐5p Exacerbated Hyperthyroidism‐Induced Liver Injury

MiR‐122 (including miR‐122‐3p and miR‐122‐5p) has been confirmed to be involved in the regulation of liver injury. However, its expression profile in hyperthyroidism‐induced liver injury models remains to be further analyzed. To this end, we used L‐Thyroxine (LT4)–induced hyperthyroidism to construct a rat model of thyroid dysfunction–induced liver injury. Compared with the sham‐operated group, the expression of miR‐122‐5p was significantly decreased in liver tissues of the LT4‐treated group, while no significant change was observed in miR‐122‐3p expression. Therefore, we subsequently focused on investigating the role and mechanism of miR‐122‐5p in hyperthyroidism‐induced liver injury (Figure S1). The abdominal dissection diagram of the rat is presented in Figure 1(a). In addition, the expression of miR‐122‐5p was also verified. The results showed that LT4 induced a significant decrease in the expression of miR‐122‐5p in the liver tissues, compared with the sham group. Furthermore, the treatment of antagomiR‐122‐5p resulted in an even greater decrease in the expression of miR‐122‐5p (Figure 1(b)). Histopathological section staining showed that in the sham group, no fatty degeneration and vacuoles were seen in the cytoplasm of the liver tissue (Figure 1(c)). However, rats with LT4‐induced hyperthyroidism exhibited cellular atrophy and a large number of fatty vacuoles in the liver tissue. Treatment with antagomiR‐122‐5p further increased cellular vacuolation and atrophy in the liver tissue (Figure 1(c)). In conclusion, the above results indicated that miR‐122‐5p deficiency aggravated liver tissue injury in hyperthyroid rats.

FIGURE 1Inhibition of miR‐122‐5p exacerbated hyperthyroidism‐induced liver injury. Male Sprague Dawley rats were divided into 3 groups: normal thyroid function group (sham), LT4‐induced hyperthyroidism group (T4 sodium), and hyperthyroidism plus miR‐122‐5p inhibitor treatment group (T4 sodium + antagomir‐122‐5P). (a) Representative photographs of the abdominal anatomy of rats in each group. (b) The mRNA expression of miR‐122‐5p in liver tissues of rats in each group was analyzed by RT‐qPCR. (c) Representative images of pathologic sections of liver tissue in each group were analyzed by H&E staining (magnification: 200x or 400x). Data are presented as mean ± SD. ^∗^ *p* < 0.05, ^∗∗^ *p* < 0.01, ^∗∗∗^ *p* < 0.001.

**Figure:**
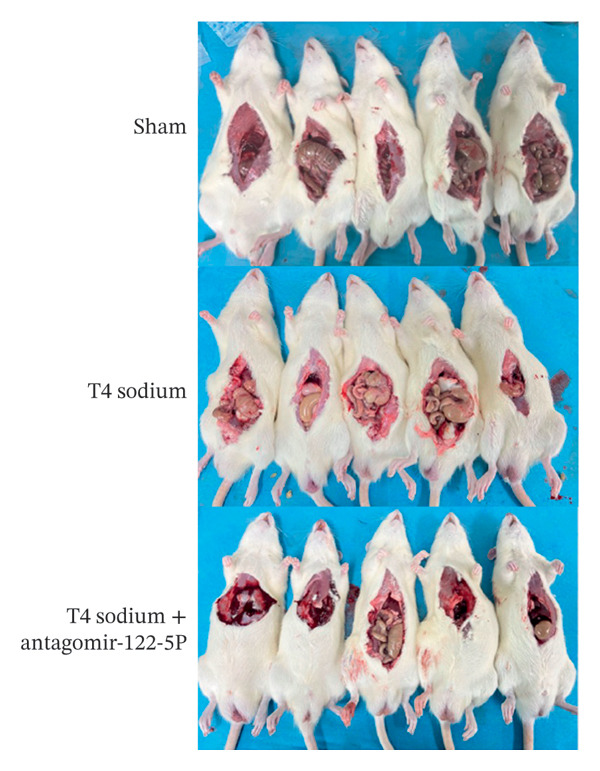
(a)

**Figure:**
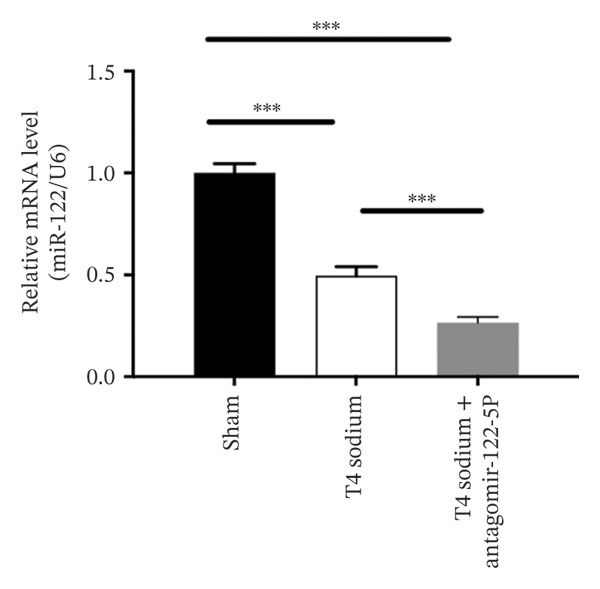
(b)

**Figure:**
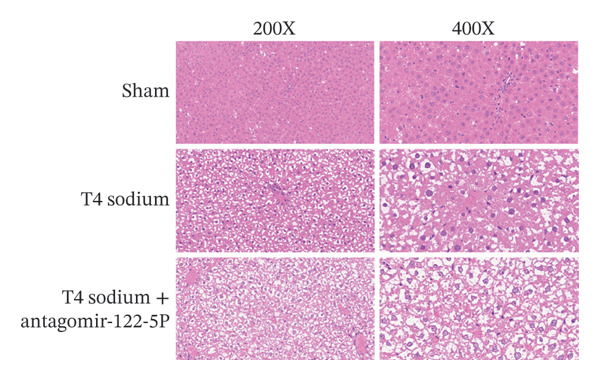
(c)

### 3.2. Inhibition of miR‐122‐5p Induced Inflammation and Ferroptosis in a Hyperthyroid Rat Model

The levels of inflammatory factors and anti‐inflammatory factors in serum were also explored by ELISA in this study. We found that LT4 increased the level of the inflammatory factor TNF‐α and decreased the level of the anti‐inflammatory factor IL‐10 (Figures 2(a) and 2(b)). However, treatment of antagomiR‐122‐5p more significantly increased the level of TNF‐α and decreased the level of IL‐10 (Figures 2(a) and 2(b)). Moreover, indicators of ferroptosis in liver tissue were analyzed. The results showed that LT4 increased iron levels in liver tissue, whereas the treatment of antagomiR‐122‐5p resulted in higher iron content in liver tissues compared to the LT4‐treated group (Figure 2(c)). WB results showed that the expression of antiferroptosis proteins, ferritin and GPX4, was significantly decreased, while the expression of ferroptosis protein ACSL4 was significantly elevated in antagomiR‐122‐5p‐treated group, compared to sham and T4 groups (Figure 2(d)). To summarize, these results suggested that miR‐122‐5p deficiency exacerbated inflammatory responses and enhanced ferroptosis in the liver tissues of hyperthyroid rats.

FIGURE 2Inhibition of miR‐122‐5p‐induced inflammation and ferroptosis in a hyperthyroid rat model. Male Sprague Dawley rats were divided into 3 groups: normal thyroid function group (sham), LT4‐induced hyperthyroidism group (T4 sodium), and hyperthyroidism plus miR‐122‐5p inhibitor treatment group (T4 sodium + antagomir‐122‐5P). The serum levels of TNF‐α (a) and IL‐10 (b) in each group of rats were analyzed by ELISA. (c) Iron levels in the liver tissue of rats in each group were analyzed by ELISA. (d) The protein expression of ferritin, GPX4, and ACSL4 in liver tissues of rats in each group was analyzed by WB. Data are presented as mean ± SD. ^∗^ *p* < 0.05, ^∗∗^ *p* < 0.01, ^∗∗∗^ *p* < 0.001.

**Figure:**
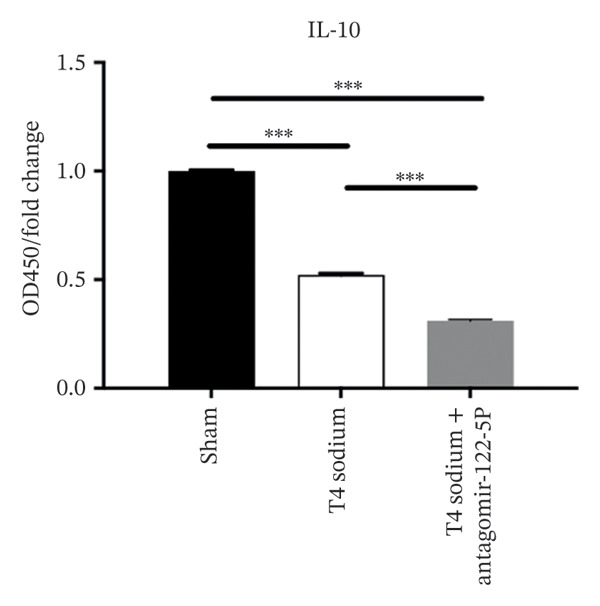
(a)

**Figure:**
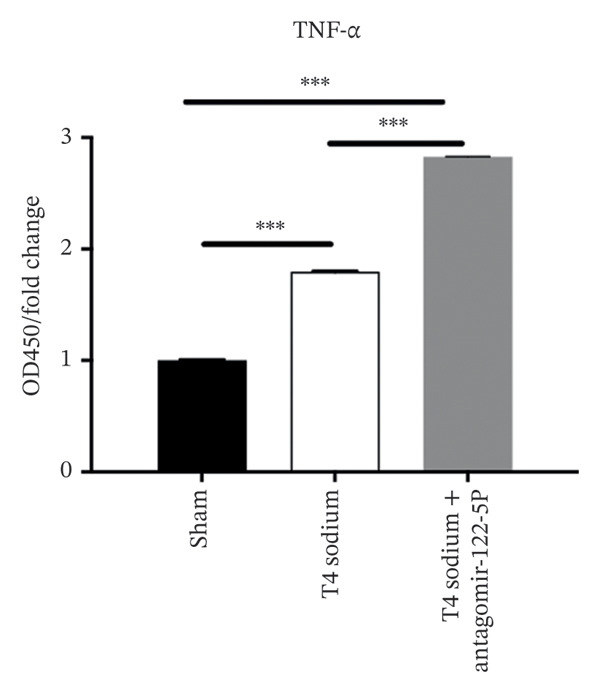
(b)

**Figure:**
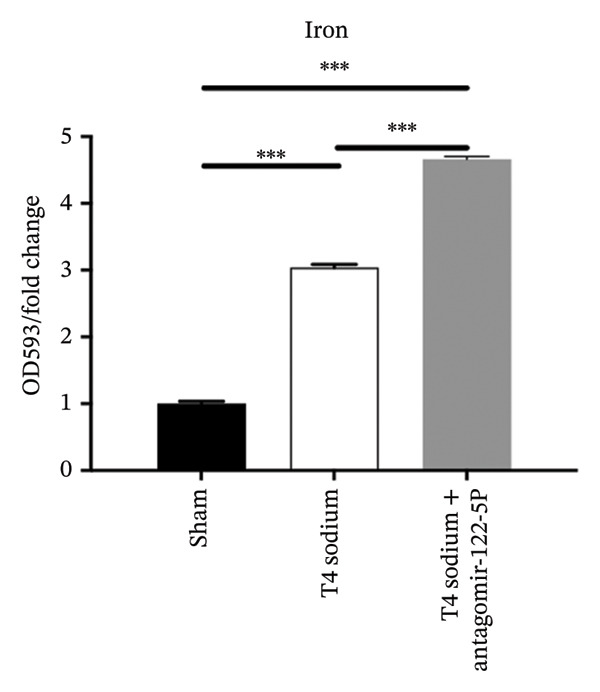
(c)

**Figure:**
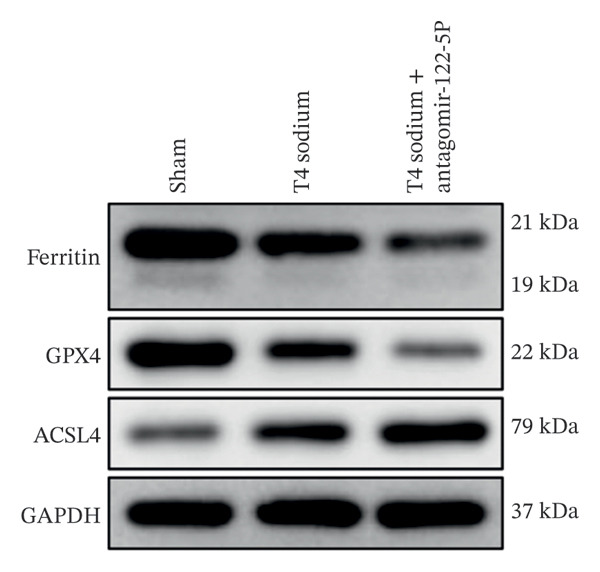
(d)

### 3.3. MiR‐122‐5p‐Targeted CCDC6

To further explore the mechanism of miR‐122‐5p, we utilized the microRNA database TargetScan and found that miR‐122‐5p could bind to the 3′‐UTR position of CCDC6, and the binding site is shown in Figure 3(a). Additionally, RT‐qPCR and WB were used to probe the expression of CCDC6, and it was found that overexpression of miR‐122‐5p inhibited the mRNA and protein expression of CCDC6 in BRL‐3A cells (Figures 3(b) and 3(c)). The dual‐luciferase reporter gene system was used to verify whether miR‐122‐5p could bind CCDC6, and the results indicated that the miR‐122‐5p mimic significantly reduced luciferase activity in 293t cells cotransfected with wild‐type CCDC6 (*p* < 0.001) but had no effect on the 3′‐UTR mutant of CCDC6 (Figure 3(d)). These results demonstrated that miR‐122‐5p downregulated CCDC6 expression by binding to the 3′‐UTR position of CCDC6.

FIGURE 3MiR‐122‐5p‐targeted CCDC6. (a) Target prediction from TargetScan indicated the 3′ UTR of CCDC6 contained putative binding sites for miR‐122‐5p. (b) The mRNA expression of CCDC6 in 293t cells was analyzed by RT‐qPCR. (c) The protein expression of CCDC6 in 293t cells was analyzed by WB. (d) Luciferase activities were measured by a dual‐luciferase reporter in 293t cells cotransfected with luciferase reporter plasmids with wild‐type 3′UTR of CCDC6 or mutant 3′UTR of CCDC6 and miR‐122‐5p mimics or NC‐mimics. Data are presented as mean ± SD. ^∗^ *p* < 0.05, ^∗∗^ *p* < 0.01, ^∗∗∗^ *p* < 0.001.

**Figure:**
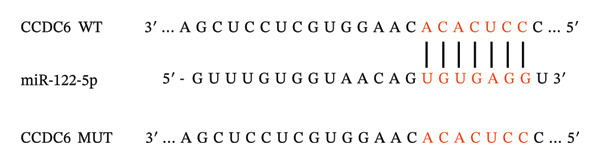
(a)

**Figure:**
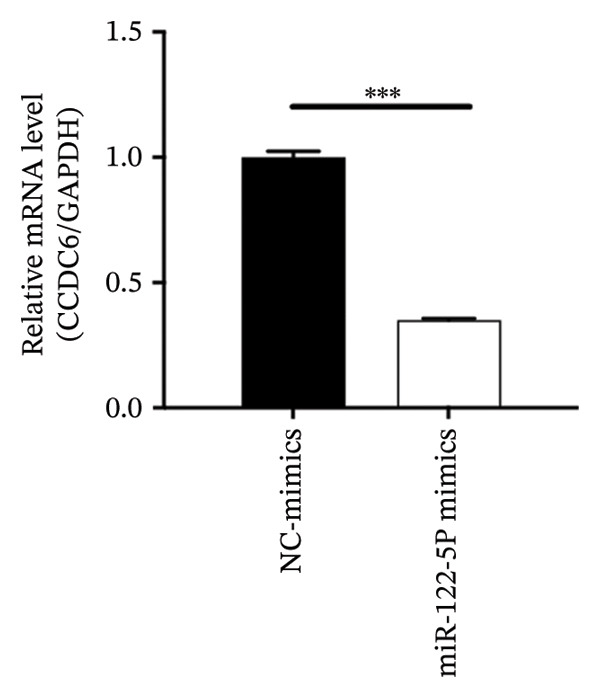
(b)

**Figure:**
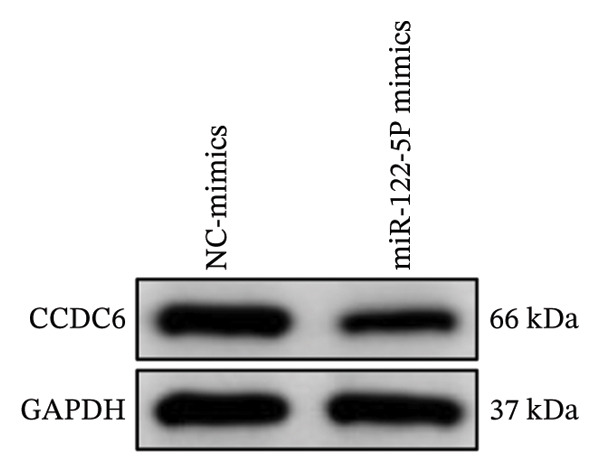
(c)

**Figure:**
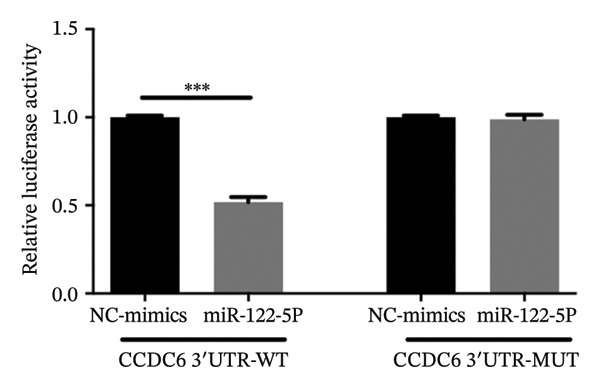
(d)

### 3.4. Inhibition of miR‐122‐5p Raised CCDC6 Expression in a Hyperthyroidism Rat Model

Whether miR‐122‐5p affects CCDC6 expression in the liver tissue of a hyperthyroid rat model was further demonstrated. IHC results of liver tissues showed that LT4 increased the expression of CCDC6 in liver tissues compared to the sham group, whereas CCDC6 expression was further elevated in a hyperthyroid rat after receiving antagomir‐122‐5p therapy (Figure 4(a)). Not surprisingly, the WB results were also consistent with the IHC results, demonstrating that inhibition of miR‐122‐5p increased CCDC6 expression *in vivo* (Figure 4(b)). The above results suggested that miR‐122‐5p negatively regulated CCDC6 expression in the liver tissue of hyperthyroid rat.

FIGURE 4Inhibition of miR‐122‐5p raised CCDC6 expression in a hyperthyroid rat model raises CCDC6 expression. Male Sprague Dawley rats were divided into 3 groups: normal thyroid function group (sham), LT4‐induced hyperthyroidism group (T4 sodium), and hyperthyroidism plus miR‐122‐5p inhibitor treatment group (T4 sodium + antagomir‐122‐5P). (a) Representative images of CCDC6 expression in liver tissues of rats from various groups were analyzed by IHC (magnification: 200 or 400). (b) The protein expression of CCDC6 in liver tissues of rats from various groups was analyzed by WB. Data are presented as mean ± SD. ^∗^ *p* < 0.05, ^∗∗^ *p* < 0.01, ^∗∗∗^ *p* < 0.001.

**Figure:**
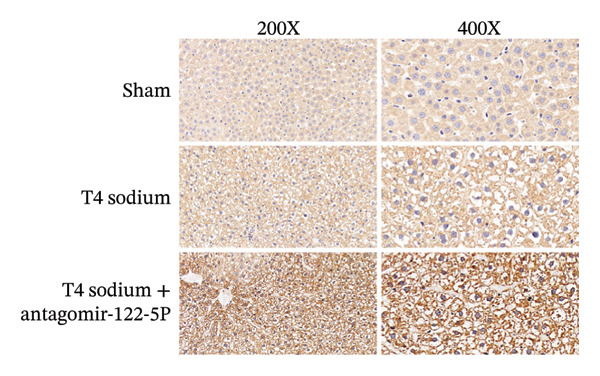
(a)

**Figure:**
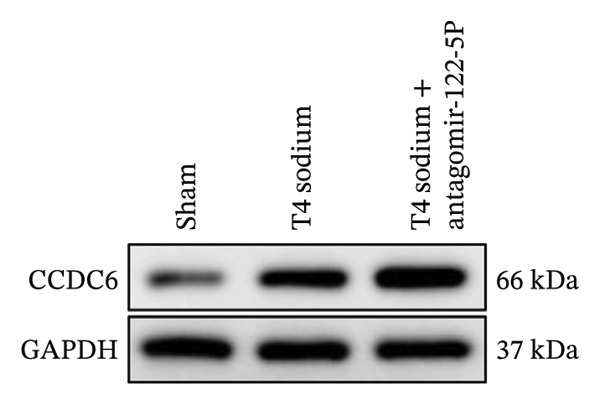
(b)

### 3.5. MiR‐122‐5p Alleviated CCL4‐Induced Ferroptosis in Hepatocytes by Regulating CCDC6 Expression

Subsequently, we further investigated the role of the miR‐122‐5p/CCDC6 axis in liver injury at the cellular level. Rat hepatocytes were treated with CCL4 to induce chronic liver injury. RT‐qPCR results showed that transfection with an miR‐122‐5p mimic upregulated miR‐122‐5p expression, while CCL4 treatment downregulated its expression (Figure 5(a)). Moreover, the miR‐122‐5p mimic restored the CCL4‐induced reduction in cell viability (Figure 5(b)). Analysis of ferroptosis‐related markers revealed that CCL4 downregulated the expression of the antiferroptosis proteins, ferritin and GPX4, while upregulating the proferroptosis protein ACSL4. These changes were reversed by the miR‐122‐5p mimic (Figure 5(c)). Furthermore, we examined the expression of CCDC6 and found that the miR‐122‐5p mimic suppressed its expression, whereas CCL4 treatment increased CCDC6 levels, indicating that miR‐122‐5p negatively regulates CCDC6 (Figure 5(d)).

FIGURE 5MiR‐122‐5p alleviated CCL4‐induced ferroptosis in hepatocytes. BRL‐3A cells were divided into 4 groups: NC group (cells transfected with NC‐mimics), miR‐122‐5p‐mimics group (cells transfected with miR‐122‐5p‐mimics), CCL4 group (treated with 50 μM CCL4), and CCL4 + miR‐122‐5p‐mimics (cells treated with CCL4 and transfected with miR‐122‐5p‐mimics). (a) The mRNA expression of miR‐122‐5p was analyzed by RT‐qPCR. (b) Cell viability was analyzed by CCK8. (c) The protein expression of ferritin, GPX4, and ACSL4 was analyzed by WB. (d) The protein expression of CCDC6 was analyzed by WB. Data are presented as mean ± SD. ^∗^ *p* < 0.05, ^∗∗^ *p* < 0.01, ^∗∗∗^ *p* < 0.001.

**Figure:**
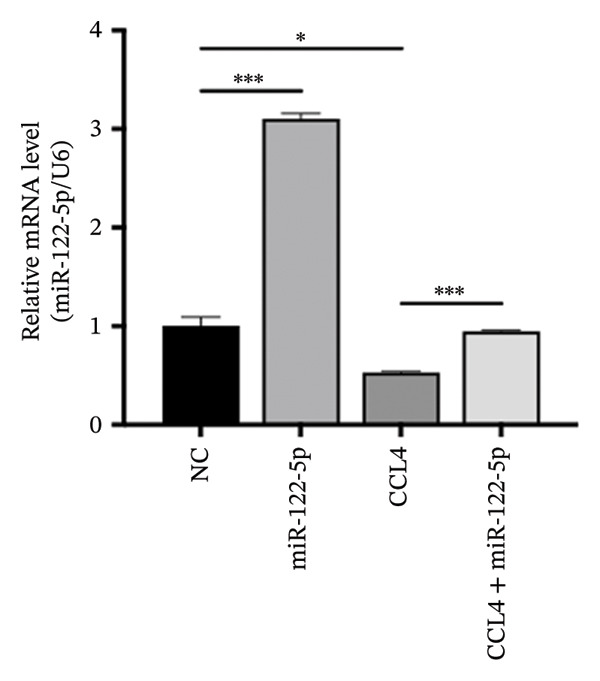
(a)

**Figure:**
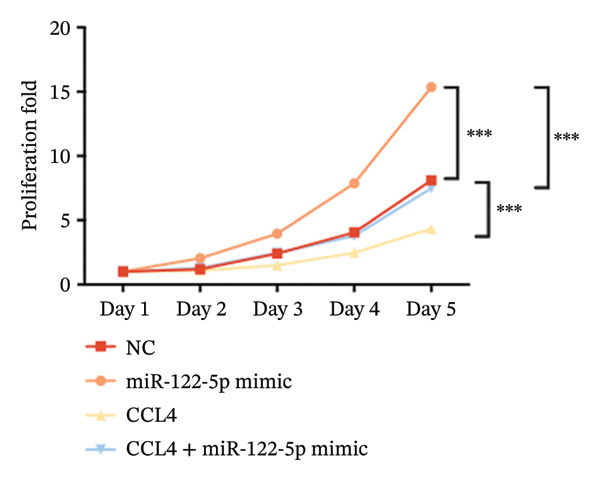
(b)

**Figure:**
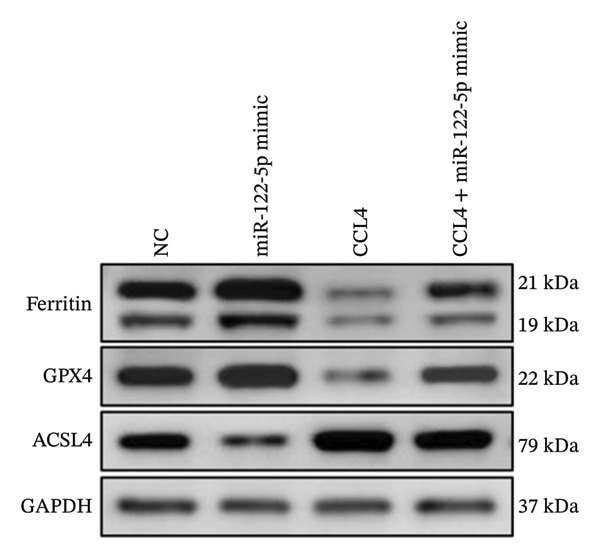
(c)

**Figure:**
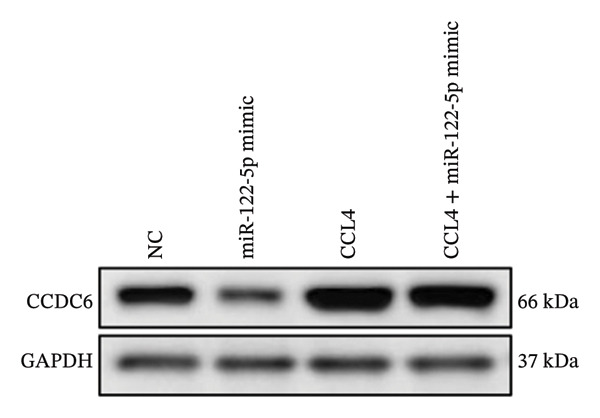
(d)

To investigate whether miR‐122‐5p exerted hepatoprotective effects through CCDC6, we introduced shRNA to knock down CCDC6 and miR‐122‐5p inhibitor in CCL4‐induced hepatocytes for rescue experiments. The results showed that the miR‐122‐5p inhibitor significantly decreased the expression of miR‐122‐5p and increased the expression of CCDC6, while the knockdown of CCDC6 reversed the effect of the miR‐122‐5p inhibitor on CCDC6 expression (Figures 6(a) and 6(b)). CCK8 and flow cytometry results showed that knockdown of CCDC6 significantly reversed miR‐122‐5p inhibitor‐induced downregulation of cell viability (Figure 6(c)) and upregulation of apoptosis (Figure 6(d)). Additionally, treatment with the miR‐122‐5p inhibitor more significantly increased the level of TNF‐α and decreased the level of IL‐10, while knockdown of CCDC6 partially reversed this result (Figures 6(e) and 6(f)). For the exploration of ferroptosis, we found that knockdown of CCDC6 inhibited the level of ferroptosis raised by the miR‐122‐5p inhibitor. On the one hand, knockdown of CCDC6 reduced intracellular iron content, and on the other hand, knockdown of CCDC6 upregulated the expression of Ferritin and GPX4, while decreasing the expression of ACSL4 (Figures 6(g) and 6(h)). Finally, hepatocytes were stained with JC‐1 to assess mitochondrial function. As shown in Figure 6(i), the miR‐122‐5p inhibitor induced an increase in green fluorescence of JC‐1 in hepatocytes, suggesting an increase in cellular damage, whereas the knockdown of CCDC6 reversed the miR‐122‐5p inhibitor‐induced cellular damage, and the fluorescence of JC‐1 showed red. To summarize, the above results demonstrated that miR‐122‐5p exerted a protective effect by inhibiting ferroptosis and mitochondrial damage in CCL4‐induced hepatocytes through negative regulation of CCDC6 expression.

FIGURE 6MiR‐122‐5p alleviated CCL4‐induced ferroptosis in hepatocytes by regulating CCDC6 expression. CCL4‐induced BRL‐3A cells were divided into 4 groups: shCtrl + NC group (cells transfected with shCtrl and NC inhibitor), shCtrl + inhibitor group (cells transfected with shCtrl and miR‐122‐5p inhibitor), shCCDC6+NC group (cells transfected with shCCDC6 and NC inhibitor), and shCCDC6+inhibitor group (cells transfected with shCCDC6 and miR‐122‐5p inhibitor). (a) The mRNA expression of miR‐122‐5p was analyzed by RT‐qPCR. (b) The protein expression of CCDC6 was analyzed by WB. (c) Cell viability was analyzed by CCK8. (d) Apoptosis was analyzed by flow cytometry. (e) The levels of TNF‐α were analyzed by ELISA. (f) The levels of IL‐10 were analyzed by ELISA. (g) Iron levels were analyzed by ELISA. (h) The protein expression of ferritin, GPX4, and ACSL4 was analyzed by WB. (i) Mitochondrial membrane potential was measured by JC‐1 staining (scale bar: 100 μm). Data are presented as mean ± SD. ^∗^ *p* < 0.05, ^∗∗^ *p* < 0.01, ^∗∗∗^ *p* < 0.001.

**Figure:**
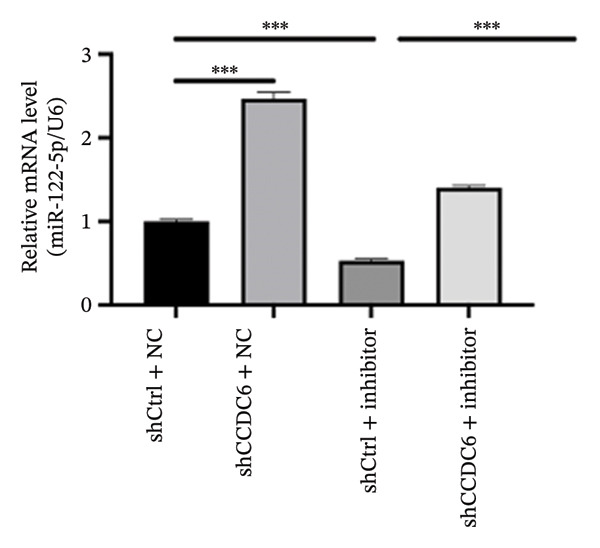
(a)

**Figure:**
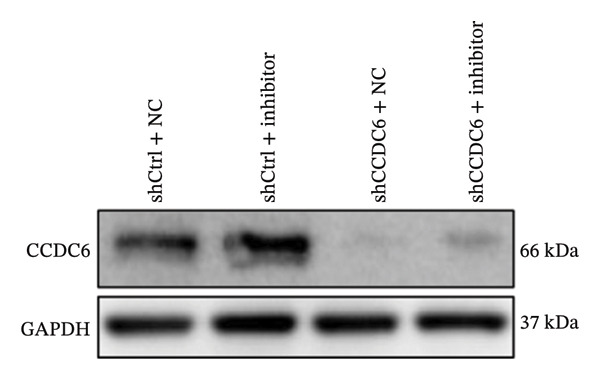
(b)

**Figure:**
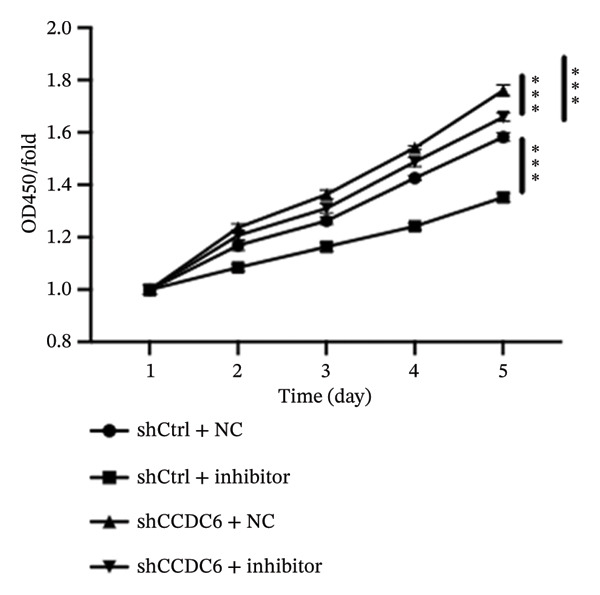
(c)

**Figure:**
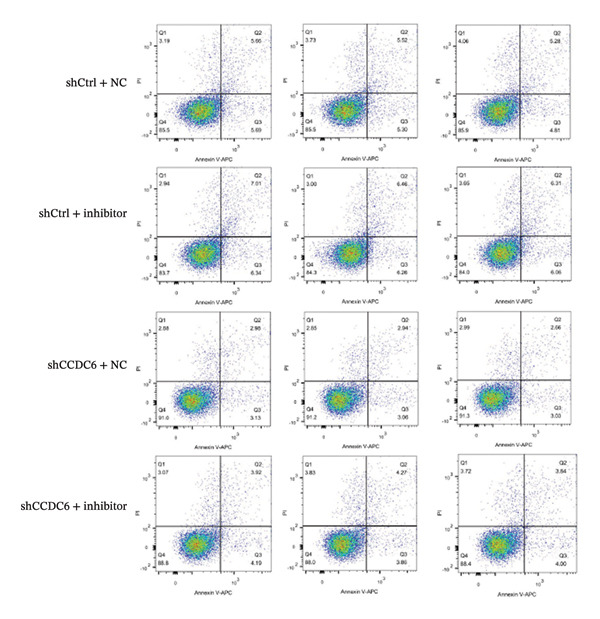
(d)

**Figure:**
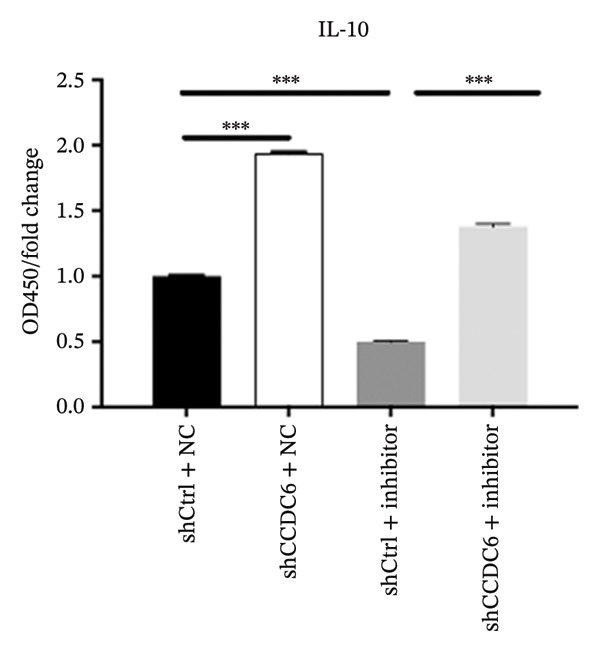
(e)

**Figure:**
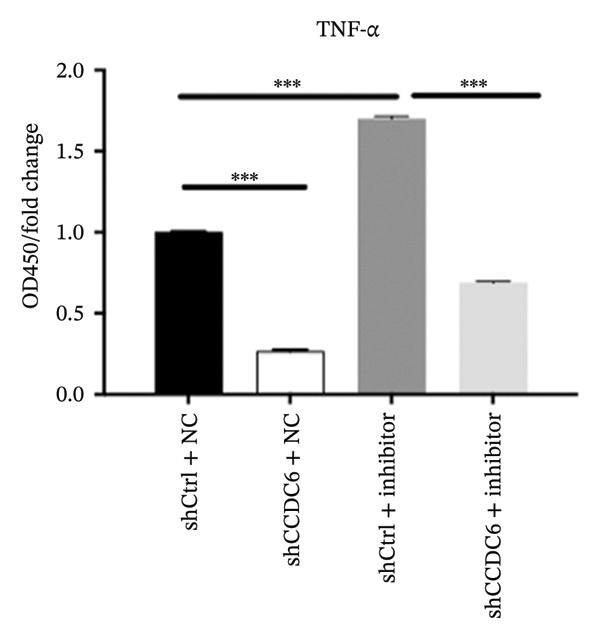
(f)

**Figure:**
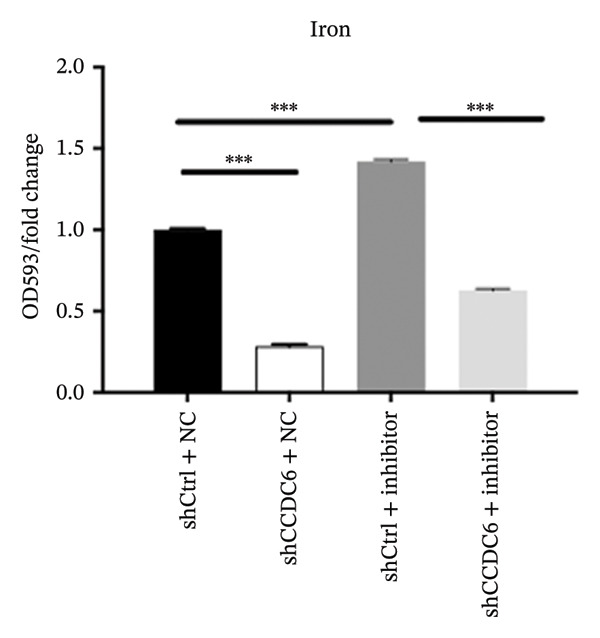
(g)

**Figure:**
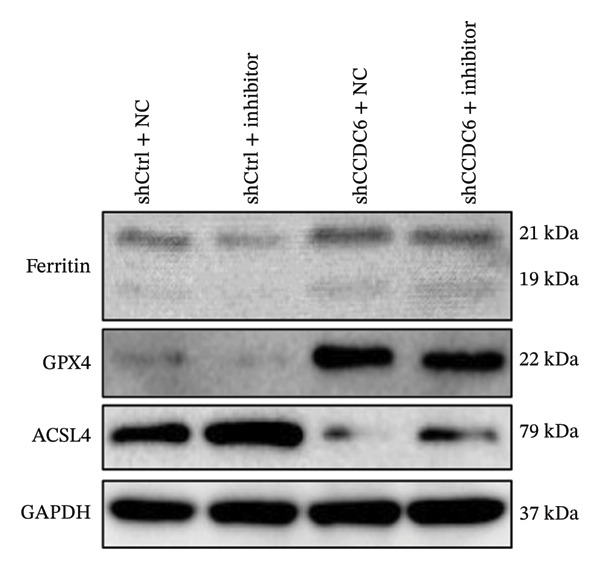
(h)

**Figure:**
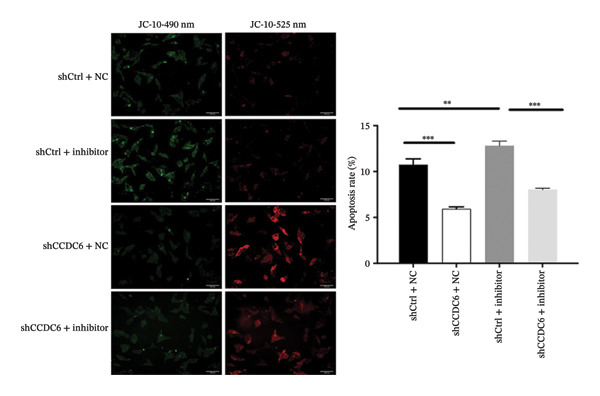
(i)

## 4. Discussion

With the development of clinical medicine and basic science research, miRNAs have received more and more attention from scholars. Currently, it has been reported in the literature that miRNAs are involved in the pathogenesis of hyperthyroidism and related organ damage diseases. Zheng et al. found that hyperthyroidism mediated a reduction in miR‐206 levels, leading to a decrease in total cholesterol (TC) and triglyceride (TG) levels to induce hepatic lipid metabolism disorders [26]. miR‐103a‐3p/TGFBR3 axis regulates TGF‐β‐induced orbital fibroblast activation and fibrosis in thyroid eye disease [27]. As a member of the miRNA family, miR‐122 is dysregulated in both thyroid diseases and liver injury. For example, miR‐122‐5p is highly expressed in GD, a thyroid disorder, suggesting its potential involvement in the pathogenesis of thyroid diseases. Recent studies have also linked miR‐221‐5p to various liver abnormalities, such as drug‐induced liver dysfunction [23, 24], while miR‐221‐3p regulates the proliferation, migration, and invasion of hepatocellular carcinoma cells by targeting LIFR [28]. However, the role of miR‐122 in hyperthyroidism‐induced liver injury remains unreported. Therefore, we investigated the expression profile of miR‐122 and found that miR‐221‐5p was upregulated in the liver tissues of LT4‐treated rats, indicating its potential involvement in liver injury. Hence, this study focuses on exploring the role and mechanism of miR‐122‐5p in regulating hyperthyroidism‐induced liver injury.

Thyroid hormones (T3 and T4) are involved in the regulation of many bodily functions, and alterations in their normal levels can lead to a number of biochemical and clinical abnormalities, such as hypothyroidism and hyperthyroidism [29, 30]. Prolonged treatment with exogenous LT4 may cause hyperthyroidism by altering thyroid activity through the induction of thyroid hormone synthesis [31]. Therefore, a large number of studies have used LT4 to construct rat hyperthyroidism models [32, 33]. In the present study, we used LT4 by gavage to construct a model of hyperthyroidism. The results revealed that miR‐122‐5p expression was reduced in the LT4‐induced rat model, suggesting that miR‐122‐5p might be closely related to hyperthyroidism. As the largest biochemical factory in the whole body, the liver is widely regulated by thyroid hormones and is one of the important target organs of the thyroid gland. Hyperthyroidism can cause liver damage, such as hepatomegaly, abnormal liver function, cirrhosis, and liver failure. Accordingly, we also explored the effect of miR‐122‐5p on liver injury. In the present study, LT‐4‐induced liver histopathological sections were similar to those previously reported in the literature [16, 34]. The results showed that LT‐4‐induced liver tissue cells atrophied and showed a large number of fatty vacuoles, suggesting that the hyperthyroidism rat liver injury model was successfully constructed. The vacuolization and atrophy of liver tissue cells further increased after MiR‐122‐5p inhibitor treatment, suggesting that miR‐122‐5p deficiency aggravated liver tissue injury in hyperthyroid rats. However, previous studies have reported conflicting views on the role of miR‐122‐5p in liver injury. For instance, in drug‐induced liver injury, loss of miR‐122‐5p has been shown to exert hepatoprotective effects [24]. Similarly, downregulation of miR‐122‐5p expression in sepsis‐induced liver injury inhibits macrophage apoptosis and alleviates liver damage [35]. The discrepancy between these findings and our results may be attributed to differences in the animal models used across studies.

It has been well documented that hyperthyroidism increases the release of inflammatory factors [36, 37]. The current study also demonstrated an increased inflammatory response in a hyperthyroid model of rats. MiR‐122‐5p deficiency exacerbated the inflammatory response. In addition, the hyperthyroid state induces high levels of ROS in liver tissue and the liver is the main storage site for iron in the body [16, 17]. The increase in iron content and ROS is a key factor in triggering the ferroptosis process [38, 39]. For this reason, we explored the changes in ferroptosis indicators in liver tissues. The results revealed that LT4 induced an increase in iron content in liver tissue and enhanced the expression of ferroptosis indicators. In addition, MiR‐122‐5p deficiency aggravated the expression of ferroptosis indicators and iron content even more, suggesting that miR‐122‐5p inhibited the ferroptosis process. This was similar to the results of previous studies. Zhao et al. found that upregulated miR‐122‐5p protected neuronal cells from cerebral hemorrhage‐induced ferroptosis [40]. Subsequently, we also explored the possible targets regulated by miR‐122‐5p. We found that miR‐122‐5p could bind CCDC6 and negatively regulate CCDC6 expression. In liver tissues of hyperthyroid rats, the expression of CCDC6 was significantly increased, and the knockdown of miR‐122‐5p increased the expression of CCDC6 even more. CCDC6 is widely reported in tumor regulation [41, 42]. Additionally, CCDC6 expression was also involved in ferroptosis. Deletion of CCDC6 could block cellular ferroptosis [43]. Meanwhile, increased ferroptosis exacerbates hepatocyte injury, thereby contributing to liver dysfunction. Furthermore, overexpression of CCDC6 has been reported to promote the malignant progression of hepatocellular carcinoma [44], indicating its detrimental role in liver tissue. Collectively, these results suggested that miR‐122‐5p might be dependent on CCDC6 expression to regulate ferroptosis processes in hyperthyroidism‐mediated liver injury.

However, this study still has limitations. miR‐122‐5p expression needs to be demonstrated in a large number of clinical samples. In addition, the molecular mechanism by which miR‐122‐5p regulates liver tissue ferroptosis needs to be refined. In addition, the current study only conducted “inhibition” (loss‐of‐function) experiments in the liver injury animal model, which cannot directly and robustly demonstrate its “protective effects.” Future studies should include miR‐122‐5p overexpression to directly confirm its therapeutic potential in the LT4‐induced model.

To summarize, this study innovatively proposed a protective role for miR‐122‐5p in hyperthyroidism‐induced liver injury. In this study, we found that inhibition of miR‐122‐5p induced an inflammatory response and ferroptosis process to aggravate hyperthyroidism‐induced liver injury. In addition, miR‐122‐5p negatively regulated the expression of CCDC6 by binding to the mRNA of CCDC6. At the cellular level, miR‐122‐5p deletion induced hepatocyte injury by exacerbating the ferroptosis process and mitochondrial damage in CCL4‐induced hepatocytes, which was dependent on the expression of CCDC6. Accordingly, the miR‐122‐5p/CCDC6 axis may serve as a potential target for the treatment of hyperthyroidism‐induced liver injury.

## Author Contributions

Guoxian Zhu: obtaining funding, data curation, experimentation, and writing of the original draft. Lidan Zhang: data curation, experimentation, and writing of the original draft. Wenbao Huang, Jiexia Ding, and Lifei Yu: data curation and experimentation. Xiangfei Xu: study conception, design, supervision, manuscript editing, and revision.

## Funding

This work was supported by the Science and Technology Program of Traditional Chinese Medicine in Zhejiang Province, Grant/Award Number: 2019ZB101.

## Disclosure

The final manuscript has been reviewed and approved by all the authors.

## Ethics Statement

All animal procedures were approved by the Affiliated Hangzhou First People’s Hospital Ethics Committee (HZSY2024033‐1) and conformed to the standards outlined in the Guide for the Ethical Care and Use of Laboratory Animals.

## Consent

All authors consent to publication.

## Conflicts of Interest

The authors declare no conflicts of interest.

## Supporting Information

Additional supporting information can be found online in the Supporting Information section.

## Supporting information


**Supporting Information 1** Supporting Figure 1: The expression of miR‐122‐5p and miR‐122‐3p in liver tissues in a hyperthyroid rat model. Male Sprague Dawley rats were divided into 2 groups: normal thyroid function group (sham) and LT4‐induced hyperthyroidism group (T4 sodium). (A) The mRNA expression of miR‐122‐5p and miR‐122‐3p in the liver tissues of rats in each group was analyzed by RT‐qPCR. Data are presented as mean ± SD. ^∗^ *p* < 0.05, ^∗∗^ *p* < 0.01, and ^∗∗∗^ *p* < 0.001.

**Supporting Information 2** Uncropped Western Blot images.

## Data Availability

The datasets used and/or analyzed during the current study are available from the corresponding author upon reasonable request.

## References

[1] Moran C. , Schoenmakers N. , Visser W. E. , Schoenmakers E. , Agostini M. , and Chatterjee K. , Genetic Disorders of Thyroid Development, Hormone Biosynthesis and Signalling, Clinical Endocrinology. (2022) 97, no. 4, 502–514, 10.1111/cen.14817. 35999191 PMC9544560

[2] Van Uytfanghe K. , Ehrenkranz J. , Halsall D. et al., Thyroid Stimulating Hormone and Thyroid Hormones (Triiodothyronine and Thyroxine): An American Thyroid Association-Commissioned Review of Current Clinical and Laboratory Status, Thyroid. (2023) 33, no. 9, 1013–1028, 10.1089/thy.2023.0169. 37655789 PMC10517335

[3] Gulcelik M. A. , Ersoz Gulcelik N. , Dinc S. , Kuru B. , Camlibel M. , and Alagol H. , The Incidence of Hyperthyroidism in Patients with Thyroid Cancer in an Area of Iodine Deficiency, Journal of Surgical Oncology. (2006) 94, no. 1, 35–39, 10.1002/jso.20508. 16788941

[4] Wiersinga W. M. , Poppe K. G. , and Effraimidis G. , Hyperthyroidism: Aetiology, Pathogenesis, Diagnosis, Management, Complications, and Prognosis, Lancet Diabetes and Endocrinology. (2023) 11, no. 4, 282–298, 10.1016/s2213-8587(23)00005-0. 36848916

[5] Vidili G. , Delitala A. , and Manetti R. , Subclinical Hyperthyroidism: the Cardiovascular Point of View, European Review for Medical and Pharmacological Sciences. (2021) 25, no. 8, 3264–3271, 10.26355/eurrev_202104_25735. 33928612

[6] Lee S. Y. and Pearce E. N. , Hyperthyroidism: A Review, JAMA. (2023) 330, no. 15, 1472–1483, 10.1001/jama.2023.19052. 37847271 PMC10873132

[7] Yorke E. , Hyperthyroidism and Liver Dysfunction: A Review of a Common Comorbidity, Clinical Medicine Insights: Endocrinology and Diabetes. (2022) 15, 10.1177/11795514221074672. PMC8829710 35153522

[8] Mansourian A. R. , Liver Functional Behavior During Thyrotoxicosis: a Review, Journal of Biological Sciences. (2013) 13, no. 8, 665–678, 10.3923/jbs.2013.665.678.

[9] Bersuker K. , Hendricks J. M. , Li Z. et al., The Coq Oxidoreductase FSP1 Acts Parallel to GPX4 to Inhibit Ferroptosis, Nature. (2019) 575, no. 7784, 688–692, 10.1038/s41586-019-1705-2. 31634900 PMC6883167

[10] Jiang X. , Stockwell B. R. , and Conrad M. , Ferroptosis: Mechanisms, Biology and Role in Disease, Nature Reviews Molecular Cell Biology. (2021) 22, no. 4, 266–282, 10.1038/s41580-020-00324-8. 33495651 PMC8142022

[11] Xing G. , Meng L. , Cao S. et al., PPARα Alleviates Iron overload-induced Ferroptosis in Mouse Liver, EMBO Reports. (2022) 23, no. 8, 10.15252/embr.202052280. PMC9346473 35703725

[12] Hirschhorn T. and Stockwell B. R. , The Development of the Concept of Ferroptosis, Free Radical Biology and Medicine. (2019) 133, 130–143, 10.1016/j.freeradbiomed.2018.09.043. 30268886 PMC6368883

[13] Wang B. , Wang Y. , Zhang J. et al., ROS-Induced Lipid Peroxidation Modulates Cell Death Outcome: Mechanisms Behind Apoptosis, Autophagy, and Ferroptosis, Archives of Toxicology. (2023) 97, no. 6, 1439–1451, 10.1007/s00204-023-03476-6. 37127681

[14] Jia F. J. and Han J. , Liver Injury in COVID-19: Holds Ferritinophagy-Mediated Ferroptosis Accountable, World Journal of Clinical Cases. (2022) 10, no. 36, 13148–13156, 10.12998/wjcc.v10.i36.13148. 36683648 PMC9850986

[15] Pan Q. , Luo Y. , Xia Q. , and He K. , Ferroptosis and Liver Fibrosis, International Journal of Medical Sciences. (2021) 18, no. 15, 3361–3366, 10.7150/ijms.62903. 34522161 PMC8436108

[16] Zhao P. , Hu Z. , Ma W. , Zang L. , Tian Z. , and Hou Q. , Quercetin Alleviates Hyperthyroidism-Induced Liver Damage via Nrf2 Signaling Pathway, BioFactors. (2020) 46, no. 4, 608–619, 10.1002/biof.1626. 32078205

[17] Chen J. , Li X. , Ge C. , Min J. , and Wang F. , The Multifaceted Role of Ferroptosis in Liver Disease, Cell Death and Differentiation. (2022) 29, no. 3, 467–480, 10.1038/s41418-022-00941-0. 35075250 PMC8901678

[18] Lu T. X. and Rothenberg M. E. , MicroRNA, The Journal of Allergy and Clinical Immunology. (2018) 141, no. 4, 1202–1207, 10.1016/j.jaci.2017.08.034. 29074454 PMC5889965

[19] Liu Y. , Wang X. , Luan W. et al., MiR-29a-3p Negatively Regulates Circulating Tfh Memory Cells in Patients With Graves’ Disease by Targeting ICOS, Immunologic Research. (2023) 71, no. 2, 173–184, 10.1007/s12026-022-09333-5. 36322282

[20] Yao X. , Wang Y. , Wang L. , Cao M. , Chen A. , and Zhang X. , Expression Patterns of Serum MicroRNAs Related to Endothelial Dysfunction in Patients With Subclinical Hypothyroidism, Frontiers in Endocrinology. (2022) 13, 10.3389/fendo.2022.981622. PMC9485940 36147570

[21] Yao Q. , Wang X. , He W. et al., Circulating microRNA-144-3p and miR-762 are Novel Biomarkers of Graves′ Disease, Endocrine. (2019) 65, no. 1, 102–109, 10.1007/s12020-019-01884-2. 30949910

[22] Singh A. K. , Rooge S. B. , Varshney A. et al., Global microRNA Expression Profiling in the Liver Biopsies of Hepatitis B virus-infected Patients Suggests Specific microRNA Signatures for Viral Persistence and Hepatocellular Injury, Hepatology. (2018) 67, no. 5, 1695–1709, 10.1002/hep.29690. 29194684

[23] Pei J. , Sun X. , Yang G. , and Zhang S. , LncRNA KCNQ1OT1 Ameliorates the Liver Injury Induced by Acetaminophen Through the Regulation of miR-122-5p/CES2 Axis, Molecular and Cellular Biochemistry. (2020) 475, no. 1-2, 107–118, 10.1007/s11010-020-03863-y. 32779042

[24] Yang Z. , Wu W. , Ou P. et al., MiR-122-5p Knockdown Protects Against APAP-Mediated Liver Injury Through up-regulating NDRG3, Molecular and Cellular Biochemistry. (2021) 476, no. 2, 1257–1267, 10.1007/s11010-020-03988-0. 33247804

[25] Cheng J. L. , Zhao H. , Yang S. G. , Chen E. M. , Chen W. Q. , and Li L. J. , Plasma miRNA-122-5p and miRNA-151a-3p Identified as Potential Biomarkers for Liver Injury Among CHB Patients with PNALT, Hepatology International. (2018) 12, no. 3, 277–287, 10.1007/s12072-018-9871-0. 29881991

[26] Zheng Y. , Zhao C. , Zhang N. et al., Serum MicroRNA miR-206 is Decreased in Hyperthyroidism and Mediates Thyroid Hormone Regulation of Lipid Metabolism in HepG2 Human Hepatoblastoma Cells, Molecular Medicine Reports. (2018) 17, no. 4, 5635–5641, 10.3892/mmr.2018.8633. 29484422 PMC5866004

[27] Xie B. , Xiong W. , Zhang F. et al., The miR-103a-3p/TGFBR3 Axis Regulates TGF-β-induced Orbital Fibroblast Activation and Fibrosis in Thyroid-Eye Disease, Molecular and Cellular Endocrinology. (2023) 559, 10.1016/j.mce.2022.111780. 36179941

[28] Tan W. , Li Z. , Xia W. , Zhu J. , and Fan R. , miR-221-3p Regulates Hepatocellular Carcinoma Cell Proliferation, Migration and Invasion via Targeting LIFR, Annals of Hepatology. (2022) 27, no. Suppl 1, 10.1016/j.aohep.2021.100567. 34699986

[29] Messarah M. , Boumendjel A. , Chouabia A. et al., Influence of Thyroid Dysfunction on Liver Lipid Peroxidation and Antioxidant Status in Experimental Rats, Experimental and Toxicologic Pathology. (2010) 62, no. 3, 301–310, 10.1016/j.etp.2009.04.009. 19540741

[30] Kundu S. , Pramanik M. , Roy S. , De J. , Biswas A. , and Ray A. K. , Maintenance of Brain Thyroid Hormone Level During Peripheral Hypothyroid Condition in Adult Rat, Life Sciences. (2006) 79, no. 15, 1450–1455, 10.1016/j.lfs.2006.04.006. 16698041

[31] Araujo A. S. , Ribeiro M. , Enzveiler A. et al., Myocardial Antioxidant Enzyme Activities and Concentration and Glutathione Metabolism in Experimental Hyperthyroidism, Molecular and Cellular Endocrinology. (2006) 249, no. 1-2, 133–139, 10.1016/j.mce.2006.02.005. 16574313

[32] Shahat A. S. , Hassan W. A. , and El-Sayed W. M. , N-Acetylcysteine and Safranal Prevented the Brain Damage Induced by Hyperthyroidism in Adult Male Rats, Nutritional Neuroscience. (2022) 25, no. 2, 231–245, 10.1080/1028415x.2020.1743917. 32264788

[33] Liu C. , Zhang Y. , Fu T. et al., Effects of Electromagnetic Fields on Bone Loss in Hyperthyroidism Rat Model, Bioelectromagnetics. (2017) 38, no. 2, 137–150, 10.1002/bem.22022. 27973686

[34] Kim S. M. , Kim S. C. , Chung I. K. , Cheon W. H. , and Ku S. K. , Antioxidant and Protective Effects of Bupleurum falcatum on the L-Thyroxine-Induced Hyperthyroidism in Rats, Evidence-Based Complementary and Alternative Medicine. (2012) 2012, 578497–12, 10.1155/2012/578497. 22888365 PMC3410357

[35] Liu S. , Xie J. , Duan C. et al., ADAR1 Inhibits Macrophage Apoptosis and Alleviates Sepsis-induced Liver Injury Through miR-122/BCL2A1 Signaling, Journal of Clinical and Translational Hepatology. (2024) 12, no. 2, 134–150, 10.14218/JCTH.2023.00171. 38343614 PMC10851074

[36] Bastawy N. , El-Mosallamy A. E. M. K. , Aljuaydi S. H. et al., SGLT2 Inhibitor as a Potential Therapeutic Approach in hyperthyroidism-induced Cardiopulmonary Injury in Rats, Pflügers Archiv. (2024) 476, no. 7, 1125–1143, 10.1007/s00424-024-02967-4. 38700719 PMC11166784

[37] Ashry M. , Askar H. , Obiedallah M. M. et al., Hormonal and Inflammatory Modulatory Effects of Hesperidin in hyperthyroidism-modeled Rats, Frontiers in Immunology. (2023) 14, 10.3389/fimmu.2023.1087397. PMC10067561 37020549

[38] Park E. and Chung S. W. , ROS-Mediated Autophagy Increases Intracellular Iron Levels and Ferroptosis by Ferritin and Transferrin Receptor Regulation, Cell Death and Disease. (2019) 10, no. 11, 10.1038/s41419-019-2064-5. PMC6817894 31659150

[39] Niu B. , Liao K. , Zhou Y. et al., Application of Glutathione Depletion in Cancer Therapy: Enhanced ROS-based Therapy, Ferroptosis, and Chemotherapy, Biomaterials. (2021) 277, 10.1016/j.biomaterials.2021.121110. 34482088

[40] Zhao H. , Li X. , Yang L. et al., Isorhynchophylline Relieves Ferroptosis-Induced Nerve Damage After Intracerebral Hemorrhage via miR-122-5p/TP53/SLC7A11 Pathway, Neurochemical Research. (2021) 46, no. 8, 1981–1994, 10.1007/s11064-021-03320-2. 33942214

[41] Wu T. , Jiang X. , Xu B. et al., High Expression of CCDC6 in Relation to Unfavorable Outcome and Immune Cells Infiltration in Hepatobiliary Carcinoma, Journal of Cancer. (2022) 13, no. 12, 3378–3395, 10.7150/jca.76050. 36186907 PMC9516016

[42] Laxmi A. , Gupta P. , and Gupta J. , CCDC6, a Gene Product in Fusion with Different Protoncogenes, as a Potential Chemotherapeutic Target, Cancer Biomarkers. (2019) 24, no. 4, 383–393, 10.3233/cbm-181601. 30909182 PMC13082534

[43] Morra F. , Merolla F. , Zito Marino F. et al., The Tumour Suppressor CCDC6 is Involved in ROS Tolerance and Neoplastic Transformation by Evading Ferroptosis, Heliyon. (2021) 7, no. 11, 10.1016/j.heliyon.2021.e08399. PMC8605351 34841108

[44] Wang J. , Xu B. , Liang L. , and Chen Q. , Long Non-coding RNA 02298 Promotes the Malignancy of HCC by Targeting the miR-28-5p/CCDC6 Pathway, Biochemical Genetics. (2024) 62, no. 6, 4967–4986, 10.1007/s10528-023-10662-9. 38381357

