# Supplementary figures and images for: Deletion of miR‐122‐5p Exacerbated Hyperthyroidism‐Induced Liver Injury by Regulating Ferroptosis
Source: Int J Endocrinol. 2026 Jun 23;2026:7047803. doi: 10.1155/ije/7047803 (PMC13287836; doi:10.1155/ije/7047803)

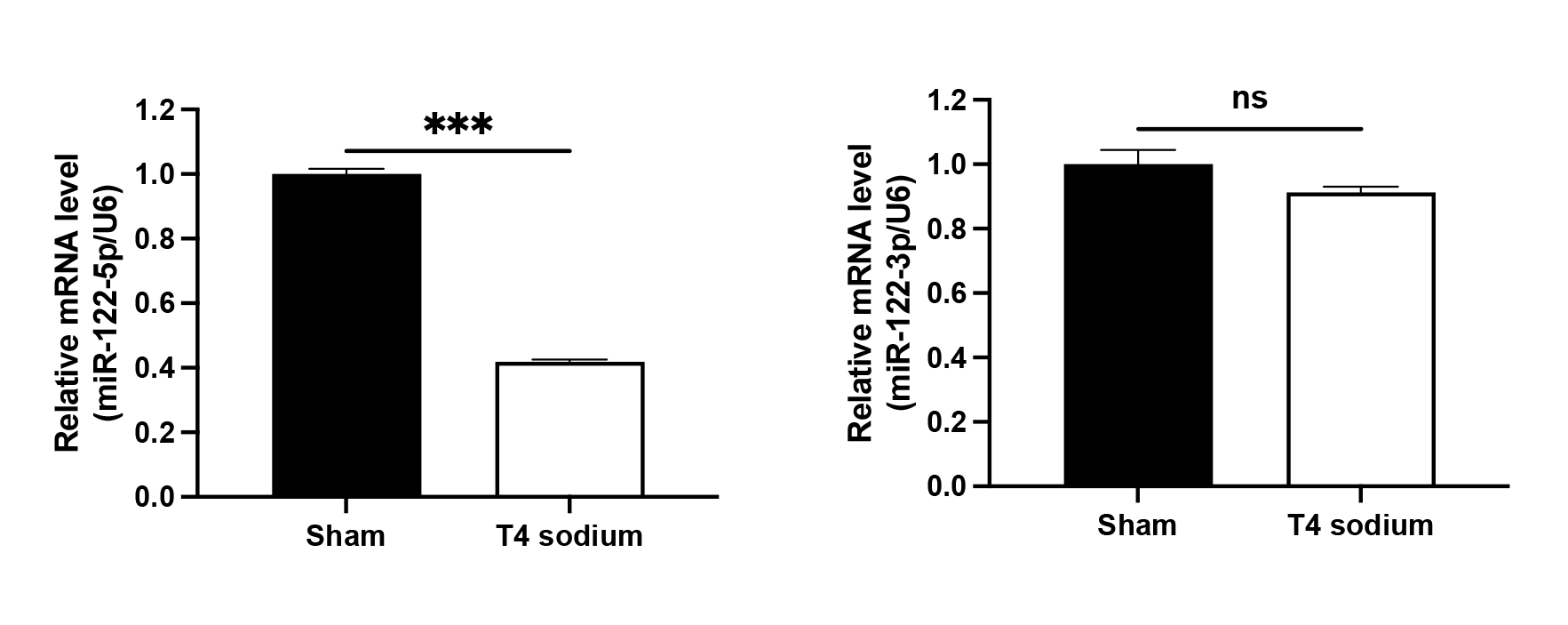

Supplement: Supplementary file 1 — Supporting Information 1 Supporting Figure 1: The expression of miR‐122‐5p and miR‐122‐3p in liver tissues in a hyperthyroid rat model. Male Sprague Dawley rats were divided into 2 groups: normal thyroid function group (sham) and LT4‐induced hyperthyroidism group (T4 sodium). (A) The mRNA expression of miR‐122‐5p and miR‐122‐3p in the liver tissues of rats in each group was analyzed by RT‐qPCR. Data are presented as mean ± SD. ∗ p < 0.05, ∗∗ p < 0.01, and ∗∗∗ p < 0.001. [file IJE-2026-7047803-s001.tif]
