# Supplementary material for: Deletion of miR‐122‐5p Exacerbated Hyperthyroidism‐Induced Liver Injury by Regulating Ferroptosis
Source: Int J Endocrinol. 2026 Jun 23;2026:7047803. doi: 10.1155/ije/7047803 (PMC13287836; doi:10.1155/ije/7047803)
Supplement: Supplementary file 2 — Supporting Information 2 Uncropped Western Blot images. [file IJE-2026-7047803-s002.docx]

**Supplementary Material 1: Uncropped Western Blot Images**

Figure 2D

Ferritin


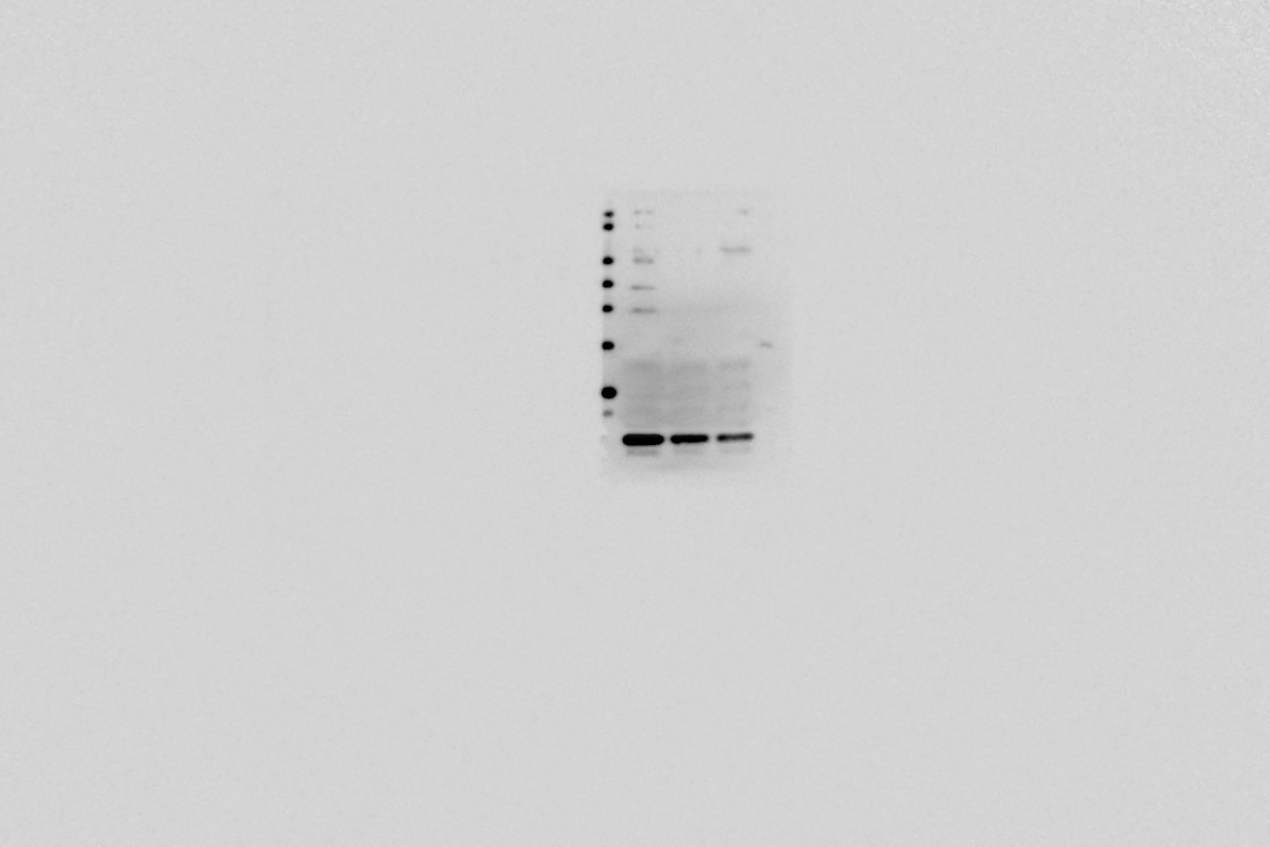


ACSL4


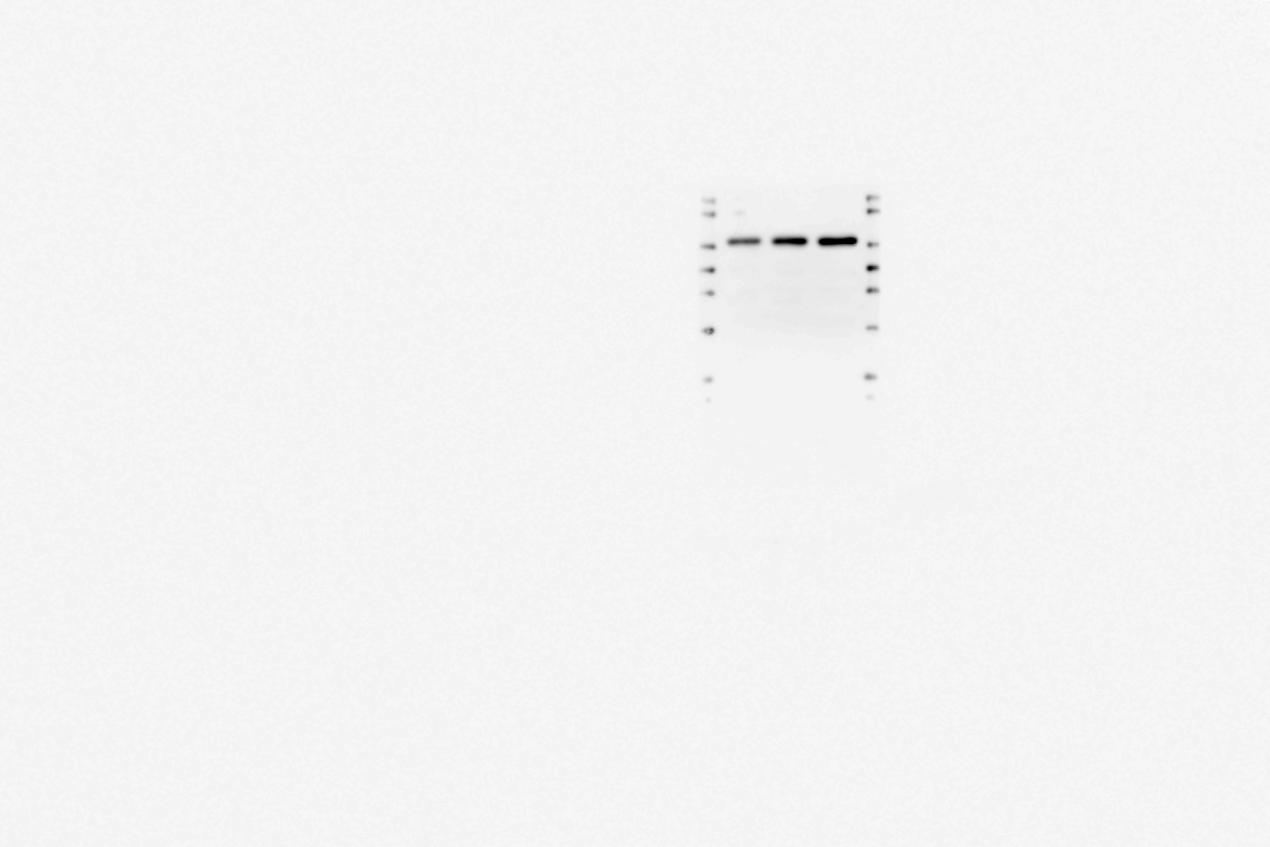


GPX4


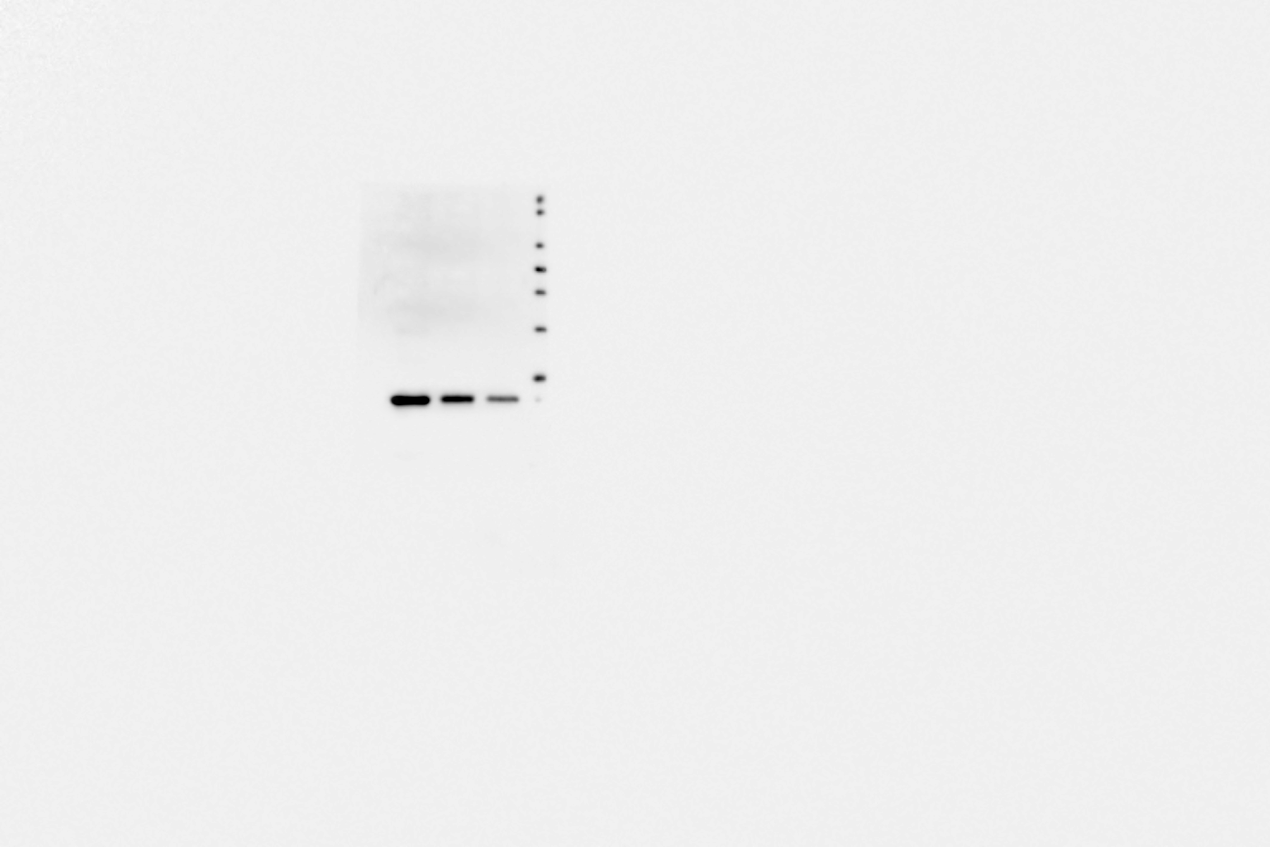


GAPDH


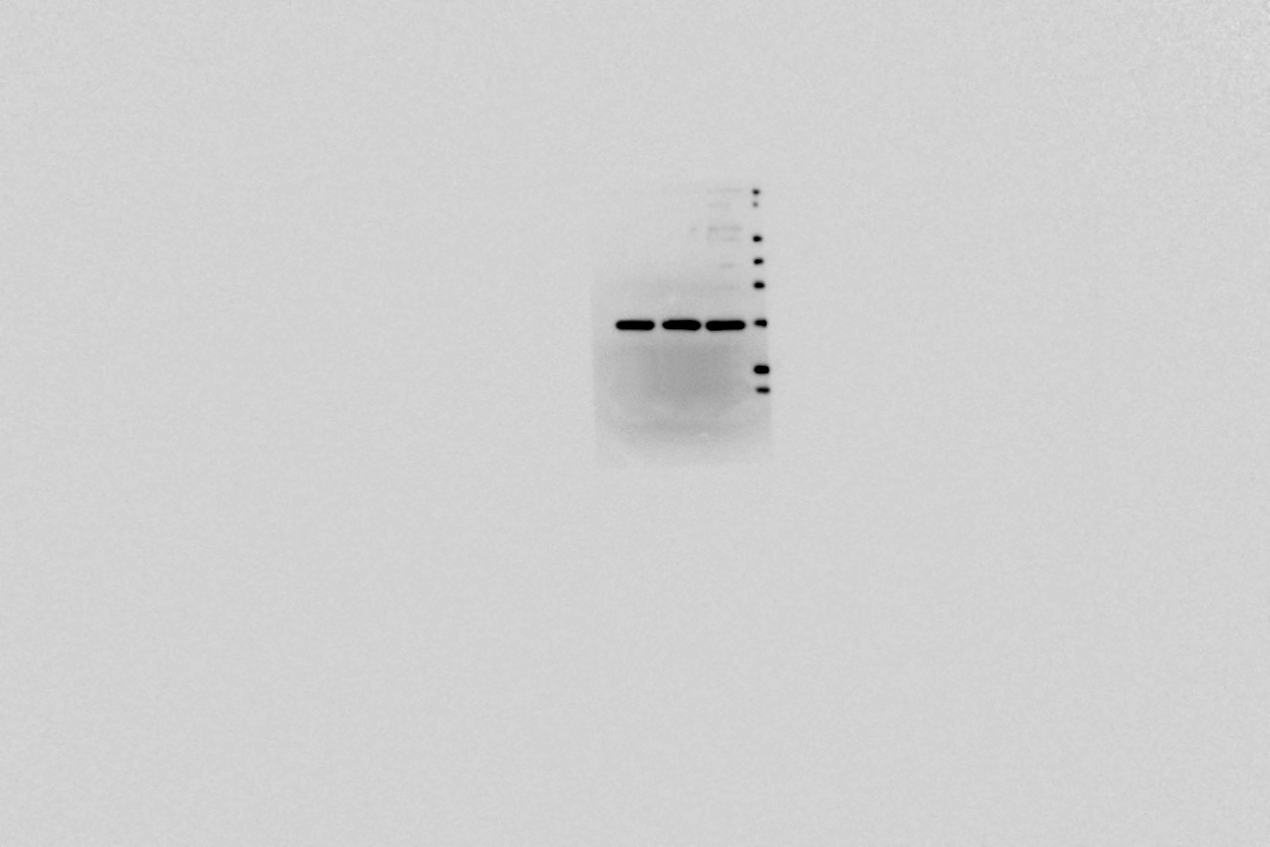


Figure 3C

CCDC6


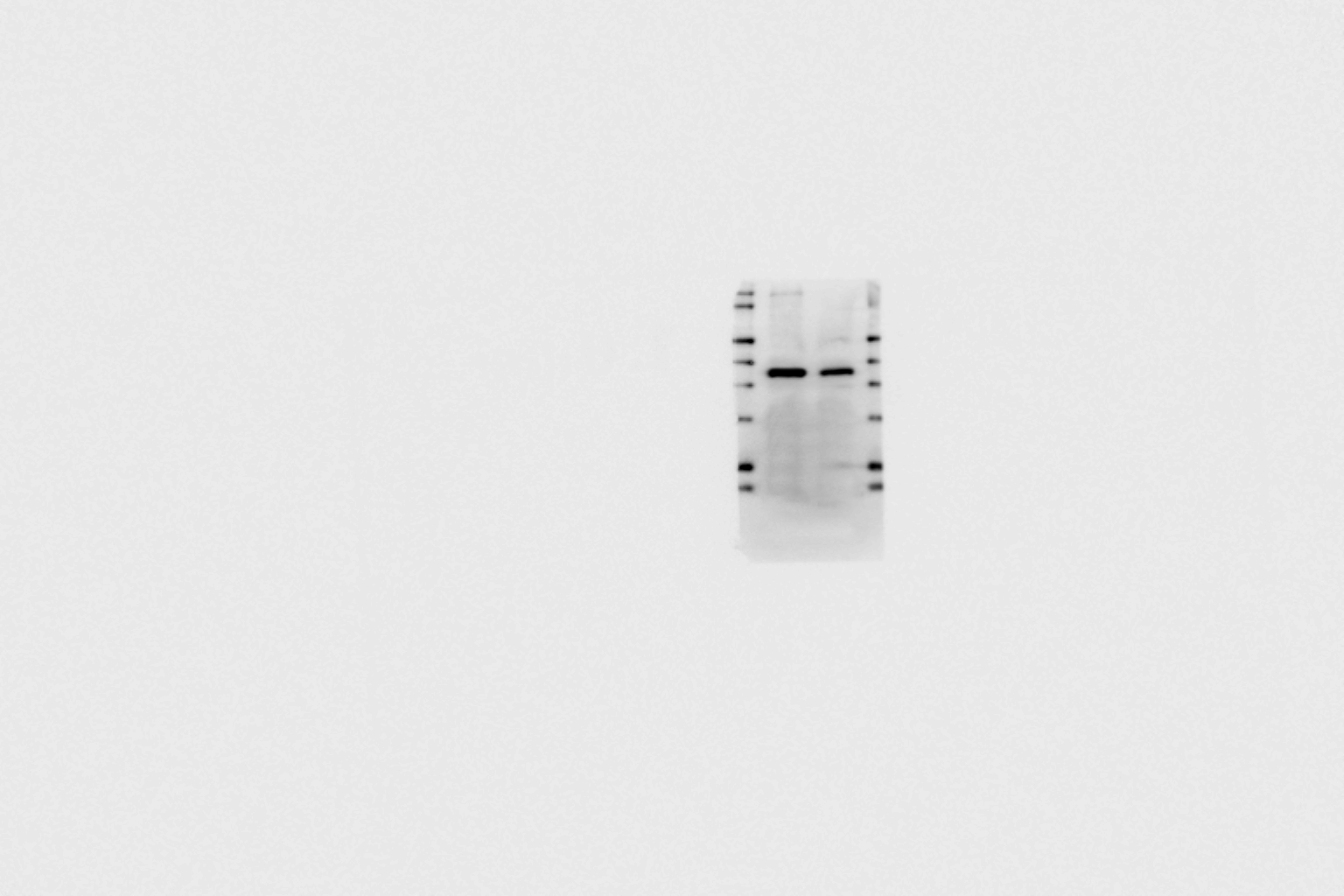


GAPDH


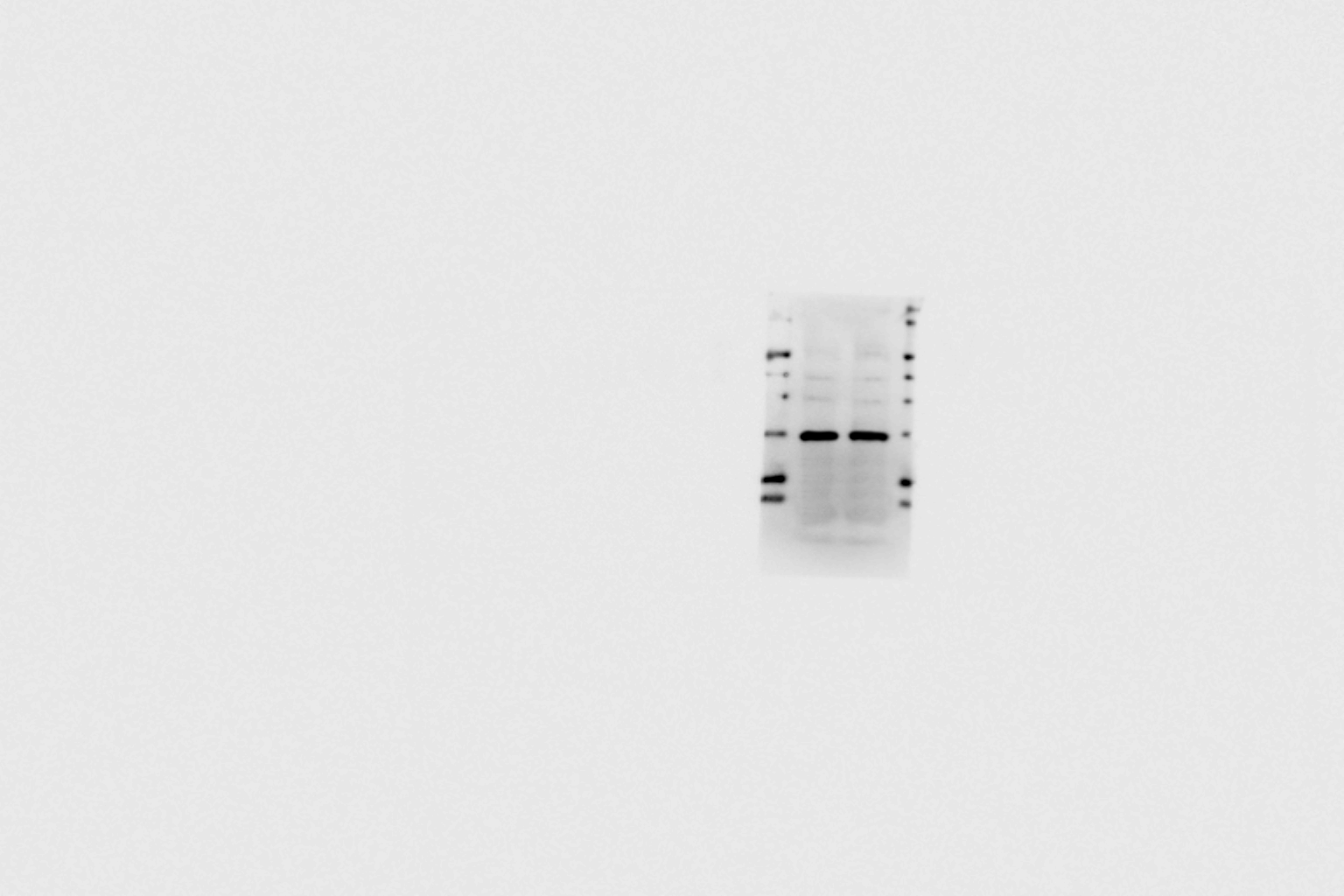


Figure 4B

CCDC6





GAPDH





Figure 5C

Feritin


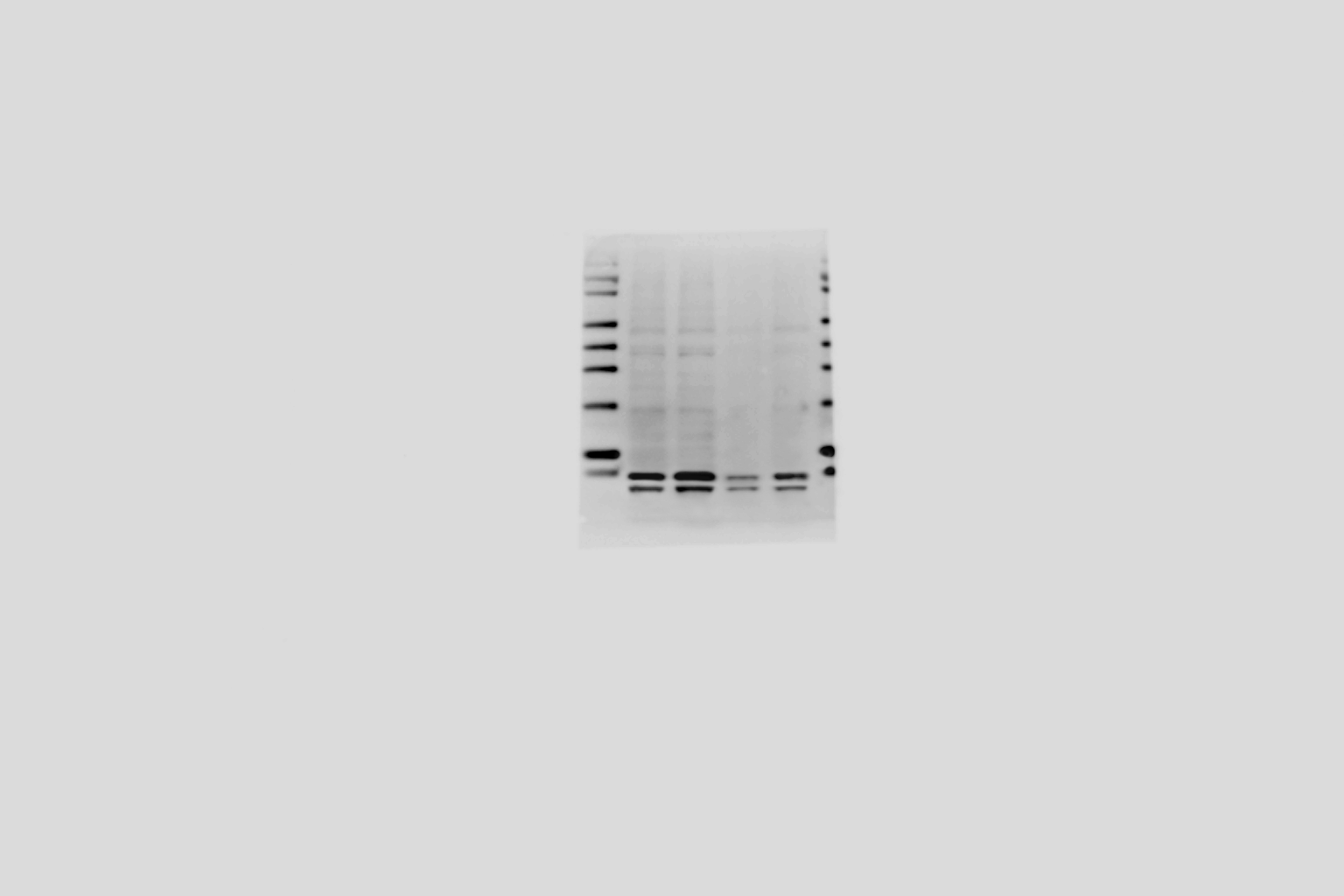


GPX4


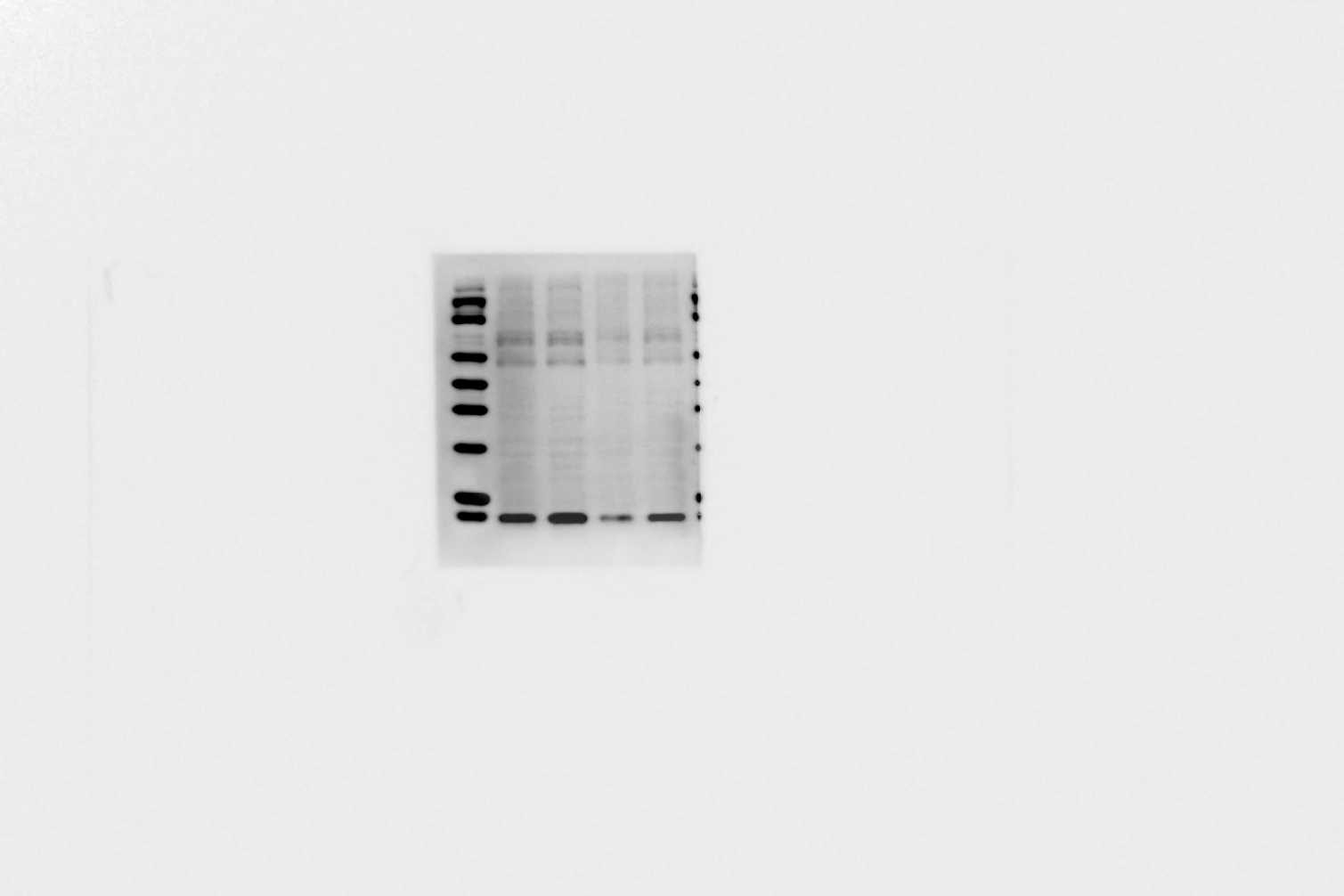


ACSL4


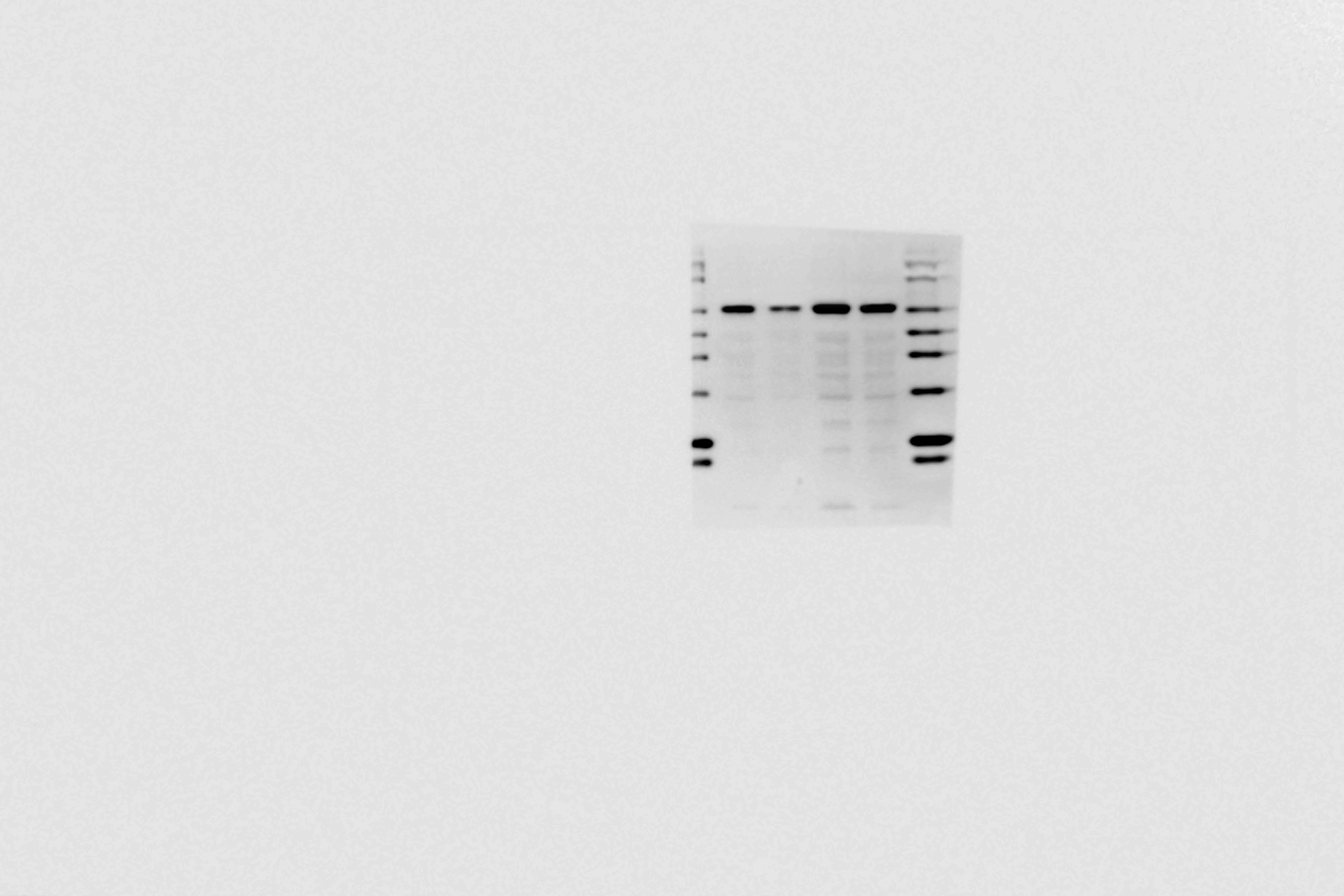


GAPDH


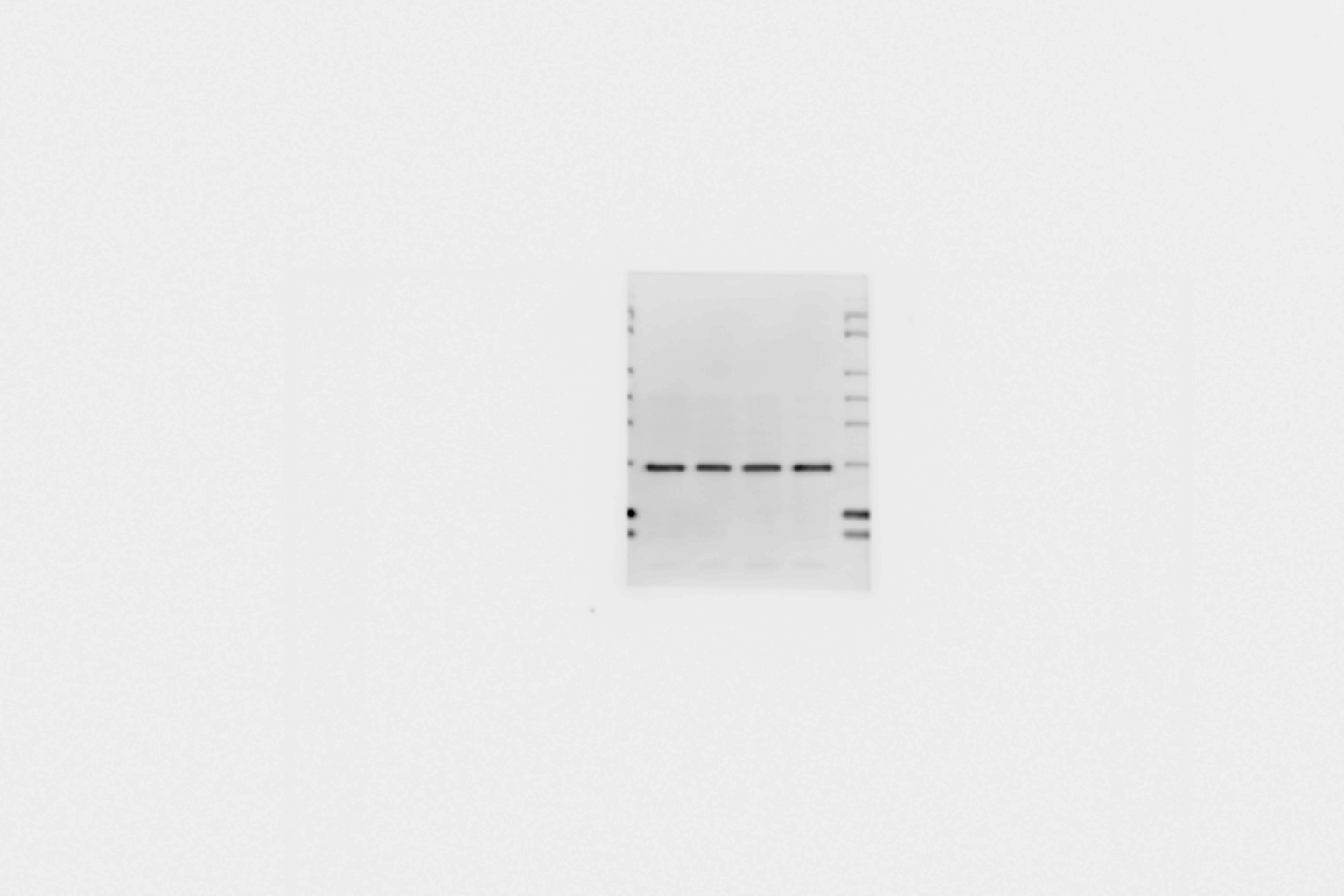


Figure 5D

CCDC6


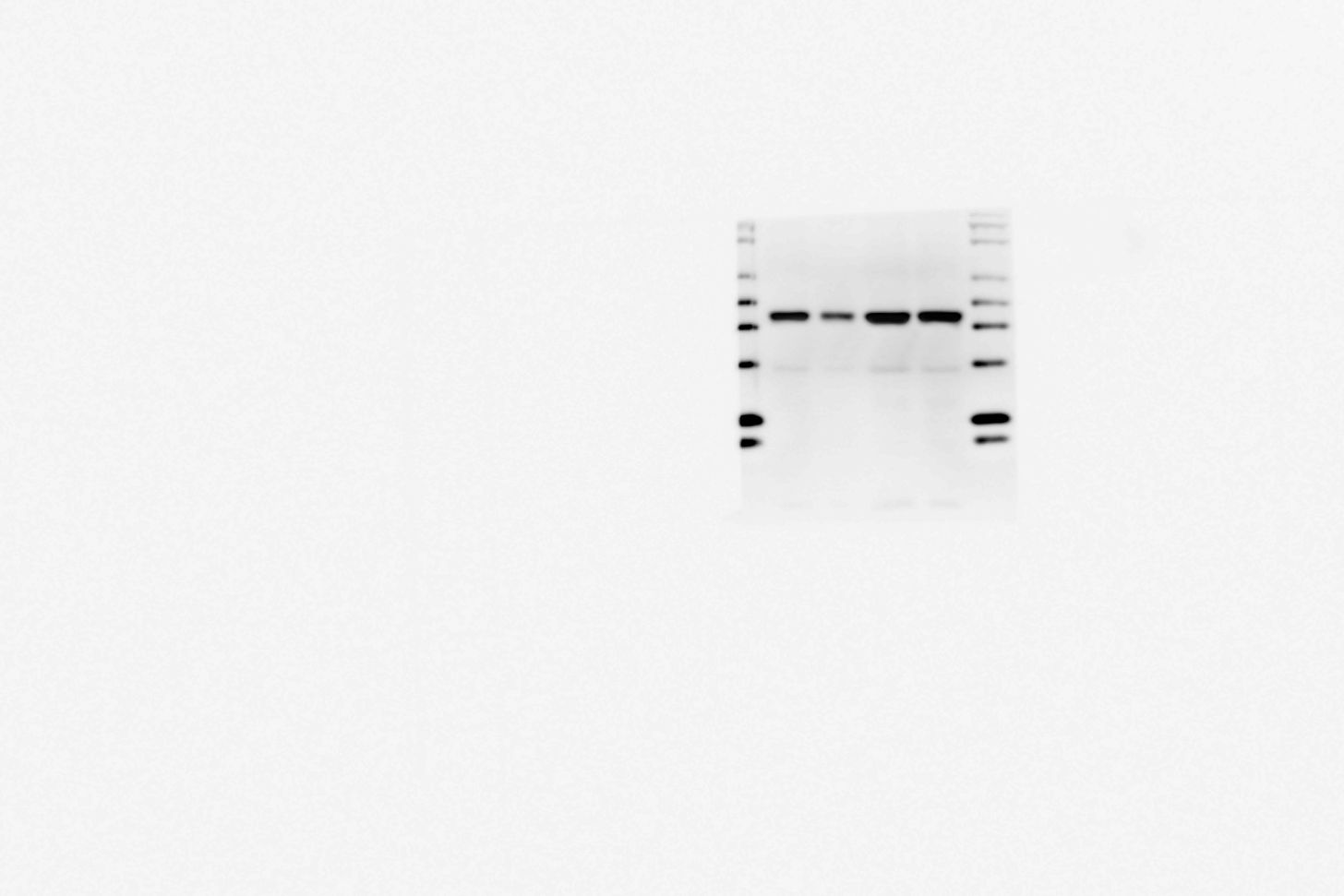


GAPDH


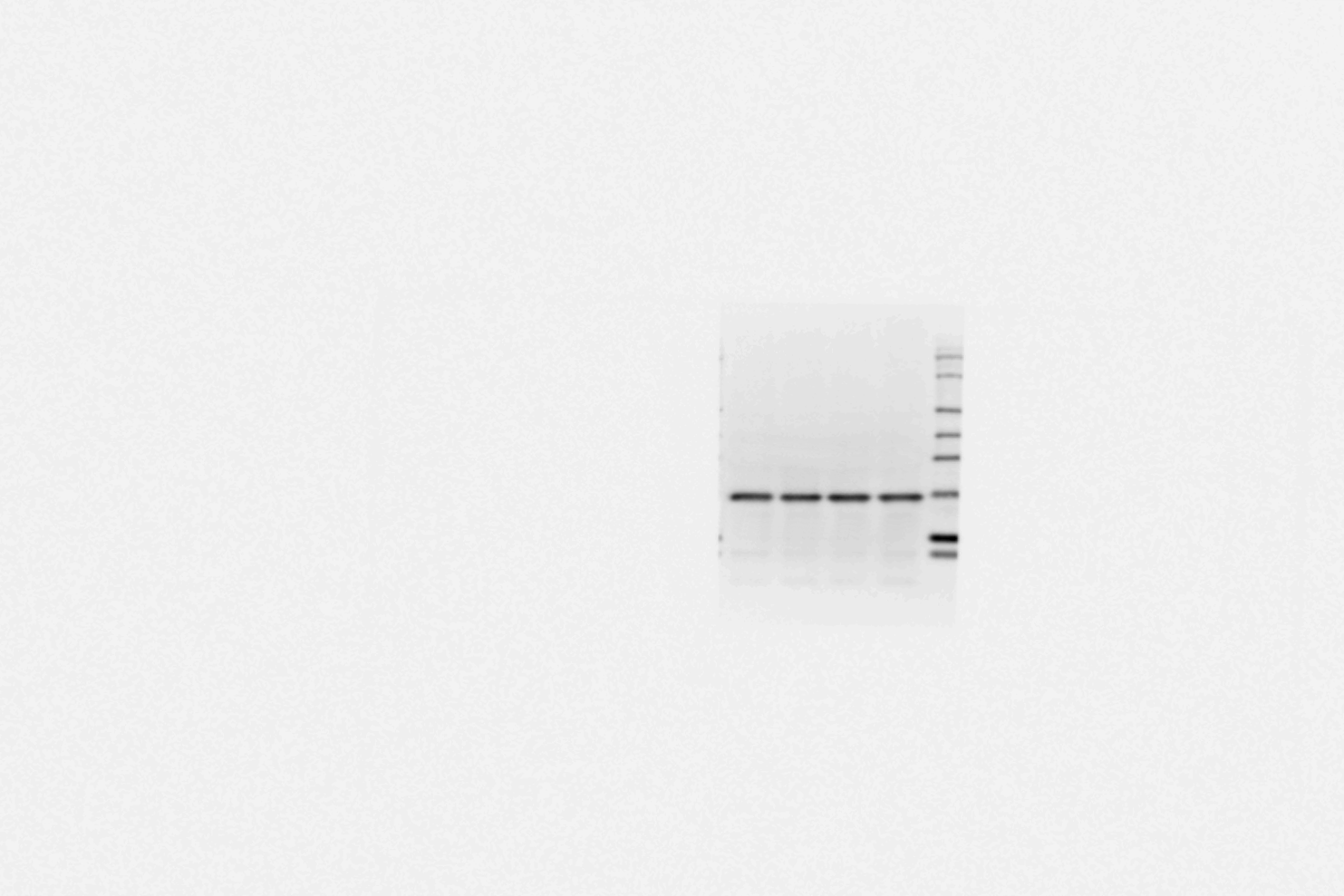


Figure 6B

CCDC6





GAPDH





Figure 6H

Ferritin


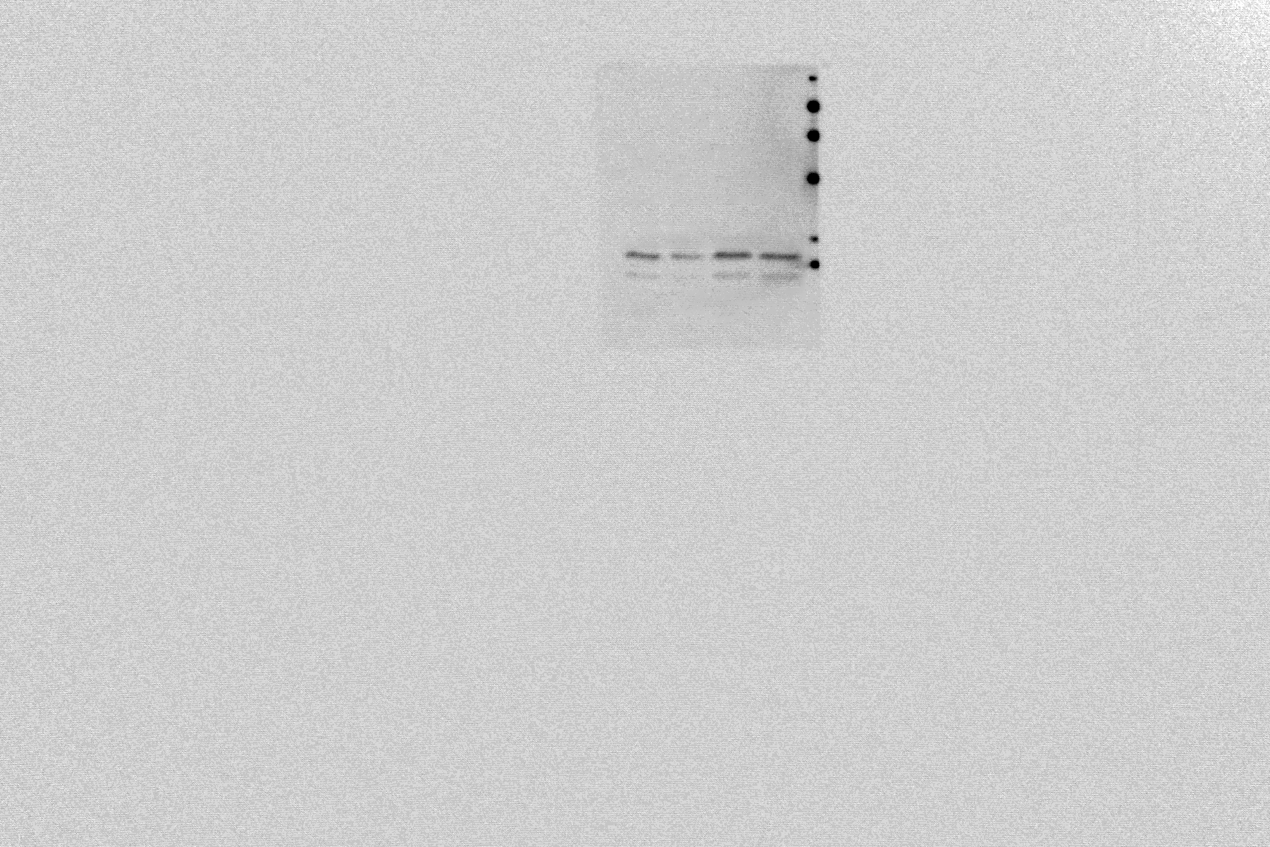


GPX4


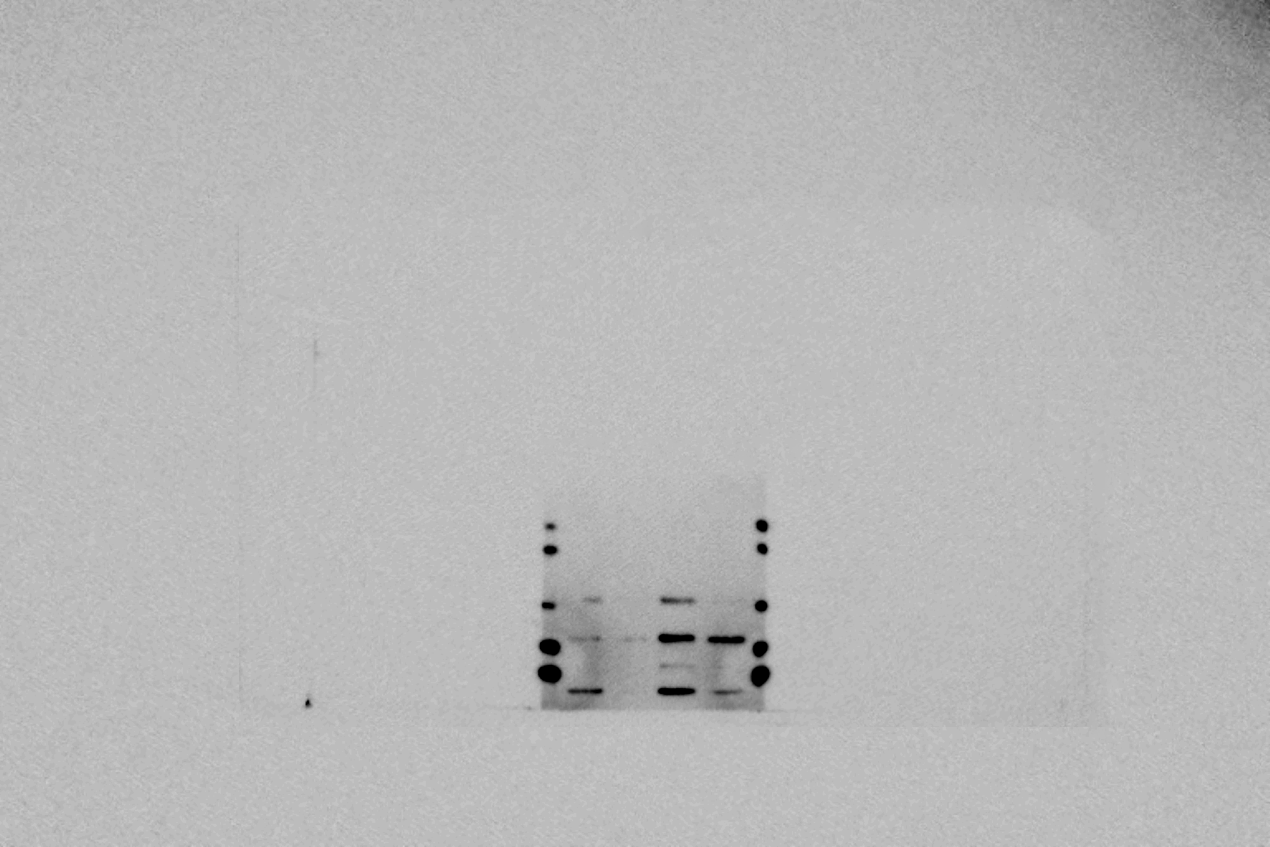


ACSL4





GAPDH
